# Efficacy and safety of Jianpi Qinghua granules for non-erosive reflux disease with spleen deficiency and damp-heat syndrome: a multicenter, randomized, double-blind, placebo-controlled clinical trial

**DOI:** 10.3389/fnut.2024.1509931

**Published:** 2025-01-07

**Authors:** Tai Zhang, Guang Bai, Wei Wang, Lin Liu, Zhenghua Zhou, Haijie Ji, Beihua Zhang, Xudong Tang

**Affiliations:** ^1^Peking University Traditional Chinese Medicine Clinical Medical School (Xiyuan), Peking University Health Science Center, Beijing, China; ^2^Peking University Health Science Center, Beijing, China; ^3^Xiyuan Hospital, China Academy of Chinese Medical Sciences, Beijing, China; ^4^Institute of Digestive Diseases, Xiyuan Hospital, China Academy of Chinese Medical Sciences, Beijing, China; ^5^Department of Gastroenterology, Affiliated Hospital of Liaoning University of Traditional Chinese Medicine, Shenyang, Liaoning, China; ^6^Department of Gastroenterology, First Teaching Hospital of Tianjin University of Traditional Chinese Medicine, Tianjin, China; ^7^Central Laboratory of Shanxi Province Academy of Traditional Chinese Medicine, Shanxi Province Academy of Traditional Chinese Medicine (Shanxi Traditional Chinese Medical Hospital), Taiyuan, China; ^8^Department of Gastroenterology, Xiyuan Hospital, China Academy of Chinese Medical Sciences, Beijing, China

**Keywords:** non-erosive reflux disease, Jianpi Qinghua granules, traditional Chinese medicine, multicenter clinical trial, randomized controlled trial, gastroesophageal reflux disease

## Abstract

**Background:**

Non-erosive reflux disease (NERD), the most frequent phenotype of gastroesophageal reflux disease, presents without visible esophageal mucosal damage but significantly impacts patients' quality of life. Current treatments like proton pump inhibitors show limited efficacy for many NERD patients, necessitating alternative approaches. Jianpi Qinghua (JQ) granules, a traditional Chinese medicine, have shown promise in treating NERD by targeting symptoms of spleen deficiency and damp-heat syndrome.

**Purpose:**

This study aims to evaluate the efficacy and safety of JQ granules in treating patients with NERD characterized by spleen deficiency and damp-heat syndrome.

**Study design:**

A multicenter, randomized, double-blind, placebo-controlled clinical trial was conducted with a total of 78 NERD patients randomly assigned to receive either JQ granules or placebo for 4 weeks, followed by a 4-week follow-up period.

**Methods:**

Seventy-eight NERD patients with spleen deficiency and damp-heat syndrome were recruited and randomly assigned to receive either JQ granules (*n* = 39) or placebo (*n* = 39). The trial included a 1-week lead-in, followed by a 4-week double-blind treatment, and a 4-week follow-up. Primary endpoints were the improvement rates of reflux and heartburn symptoms and VAS score changes. Secondary endpoints included atypical symptom scores, total TCM syndrome scores, GERD Health-Related Quality-of-Life (HRQL), and self-rated depression and anxiety scales. Safety assessments involved routine blood, urine, and liver and kidney function tests.

**Results:**

After 4 weeks, the improvement rate for reflux or heartburn symptoms was 79.49% in the JQ group vs. 58.97% in the placebo group (*P* < 0.05). VAS scores showed significant reductions in both groups but without notable inter-group differences. Total TCM syndrome scores significantly decreased in both groups, with the JQ group showing greater improvement trends. The JQ group had higher rates of effective TCM syndrome improvement and better GERD-HRQL scores. Both groups saw significant reductions in self-rated depression and anxiety scores, with trends favoring JQ granules. Safety assessments were comparable between groups.

**Conclusion:**

JQ granules significantly outperform placebo in treating NERD symptoms and display long-term effectiveness. They effectively address spleen deficiency and damp-heat syndrome, improving patients' social functioning, and have a favorable safety profile.

**Clinical trial registration:**

https://clinicaltrials.gov/study/NCT04324138?term=NCT04324138&rank=1, identifier: NCT04324138.

## Introduction

GERD refers to a condition where gastric-duodenal contents reflux, leading to a range of symptoms and/or complications (1). Typically, GERD is categorized into reflux esophagitis (RE), identified by mucosal damage visible during endoscopy, and non-erosive reflux disease (NERD), which lacks such damage and represents about 70% of GERD cases (2). Some researchers argue that Barrett's esophagus should also be considered a complication of GERD (1). In severe cases of RE, particularly classified as grade C/D according to the Los Angeles criteria, key characteristics include abnormal esophageal acid exposure primarily due to hiatal hernia and impaired esophageal peristalsis, coupled with significant dysfunction of the gastroesophageal junction anti-reflux barrier (3). Therefore, treatment goals for RE patients emphasize curing complications, preventing symptom and complication recurrence, correcting anatomical abnormalities, restoring anatomical structure and function, and normalizing abnormal reflux patterns to reduce inflammatory damage (4).

Drug therapy serves as the primary treatment for GERD. Currently, due to the absence of effective targeted medications for harmful substances in reflux beyond acid (such as gastric proteases, pancreatic proteases, bile, etc.), proton pump inhibitors (PPIs) and potassium-competitive acid blockers (P-CABs) are recommended as the initial therapeutic choices. Both classes of drugs suppress gastric acid secretion effectively. For patients with RE, a standard daily dose of PPIs over 4 weeks achieves complete symptom relief in 70%-80% of cases (5). Extending treatment to 8 weeks can further promote esophageal mucosal healing, reaching rates between 85% and 96% (6).

Since NERD patients lack endoscopic esophageal mucosal damage, and only 4.4% of NERD cases naturally progress to RE (7), their treatment goals differ from those with RE, primarily focusing on symptom relief and improving patient quality of life. However, standard-dose PPI therapy once daily for 4 weeks achieves symptom relief in 51% of NERD patients (8). Doubling the PPI dosage shows no significant difference in relieving heartburn symptoms compared to standard-dose therapy (*P* = 0.27) (9), with 45% of NERD patients still showing poor or no response to the higher dosage (10). Therefore, compared to RE, NERD patients are more likely to develop resistance to acid suppression therapy, manifested as refractory GERD (rGERD).

The pathogenesis of NERD differs significantly from that of RE and encompasses a highly heterogeneous population. According to the latest Rome IV criteria from 2016, heartburn-related diseases are classified into four categories: RE, NERD, reflux hypersensitivity, and functional heartburn (11). In clinical practice, NERD broadly refers to conditions where endoscopy does not reveal esophageal mucosal damage, including true NERD with abnormal esophageal acid exposure, reflux hypersensitivity characterized by heightened sensitivity to both physiological acid and non-acid reflux despite normal acid load, and functional heartburn where symptoms are unrelated to reflux (12). Thus, factors beyond gastric acid reflux—such as non-acid reflux, gas reflux, and the presence of digestive enzymes, bile, and chyme in refluxate—are significant contributors to the pathogenesis or exacerbation of NERD symptoms (13, 14). While acid suppression therapy effectively relieves symptoms in half of NERD patients, highlighting its importance and efficacy for some, focusing solely on acid suppression overlooks other components of reflux events, thus limiting the management of symptoms.

Jianpi Qinghua (JQ) granules, an empirical Traditional Chinese Medicine (TCM) formula often used to treat gastrointestinal disorders, particularly GERD. Zhang et al. (15) conducted a multicenter, randomized, double-blind, double-dummy, noninferiority study to evaluate the efficacy of JQ granules combined with a reduced dose of omeprazole in treating NERD. JQ granules were administered to 204 patients alongside a lower dose of omeprazole (10 mg) compared to a control group receiving the standard omeprazole dose (20 mg). The study found that the combination therapy resulted in a higher rate of complete resolution of symptoms (40.8% vs. 26.8%) and increased gut microbiota diversity (15). Additionally, the combination therapy corrected the glutamate metabolism pathway, potentially alleviating anxiety and visceral hypersensitivity associated with NERD (15). The study concludes that JQ granules combined with a reduced dose of omeprazole are more effective and better tolerated than the standard omeprazole dose, offering a promising alternative treatment strategy for NERD (15).

This study builds on previous investigations into the efficacy of JQ granules in treating NERD. While prior research demonstrated the superior efficacy of JQ granules combined with a reduced dose of omeprazole in general NERD patients (15), the heterogeneity of NERD necessitates a more tailored therapeutic approach. TCM emphasizes the importance of syndrome differentiation, and spleen deficiency with dampness-heat syndrome is a common subtype in NERD patients (16, 17). This subgroup is characterized by hallmark symptoms, including postprandial acid reflux, bloating, burning pain in the upper abdomen, and loose and sluggish stool (16). Targeting this specific subgroup enables a focused evaluation of JQ granules in addressing both typical and atypical NERD symptoms, while simultaneously treating the underlying TCM syndrome.

The composition of JQ granules reflects their design to address the unique challenges of spleen deficiency and dampness-heat syndrome in NERD patients. Key ingredients, such as Codonopsis pilosula and Atractylodes lancea, are traditionally used to strengthen the spleen and resolve dampness, while Scutellaria baicalensis and Coptis chinensis target damp-heat. Additionally, Sepiella maindroni contributes to the neutralization of stomach acid, aligning with modern understandings of NERD pathophysiology. This tailored formulation ensures a holistic approach to managing gastrointestinal symptoms and addressing the broader syndrome pattern, integrating TCM principles with evidence-based clinical practice.

This study was designed to assess the efficacy of JQ granules for NERD patients characterized by spleen deficiency and dampness-heat syndrome. Primary objectives included evaluating improvements in key symptoms such as reflux and heartburn, tracking changes in both typical and atypical symptom scores, and measuring health-related quality of life using the Gastroesophageal Reflux Disease-Health-Related Quality of Life (GERD-HRQL) scale. Safety assessments were integral to the study design, providing robust evidence of the treatment's efficacy and safety profile.

## Materials and methods

### Study design and participants

This study is a multicenter, randomized, double-blind, placebo-controlled clinical trial conducted across three institutions: Xiyuan Hospital of the China Academy of Chinese Medical Sciences, the Affiliated Hospital of Liaoning University of Traditional Chinese Medicine, and the First Affiliated Hospital of Tianjin University of Traditional Chinese Medicine. A total of 78 patients diagnosed with NERD characterized by spleen deficiency and damp-heat syndrome were enrolled in the trial. Participants were randomly assigned to either the experimental group or the control group, with each group consisting of 39 individuals.

To minimize heterogeneity across the three sub-centers, several measures were implemented: (1) standardized protocols: a unified trial protocol detailing patient recruitment, diagnostic criteria, randomization, treatment administration, and outcome assessments was followed at all centers; (2) investigator training: all TCM practitioners and clinical investigators underwent centralized training sessions led by the principal investigator. These sessions emphasized consistency in diagnosis, treatment, and data collection processes; (3) harmonized interventions: The same batch of JQ Granules and placebo was used at all centers, ensuring uniform quality and preparation of interventions; (4) blinded outcome assessment: all outcome assessments were conducted by investigators blinded to group allocation, reducing subjective bias across centers; (5) centralized data management: all data from the sub-centers were collected and managed in a centralized database, with periodic audits conducted to ensure data consistency and integrity.

The trial design encompassed a 1-week run-in period, followed by a 4-week double-blind treatment phase, and concluded with a 4-week follow-up period. All participants provided informed consent prior to enrollment, adhering to ethical standards.

During the run-in period, patients were assessed for eligibility and prepared for the treatment phase. The specific activities during this period included: (1) washout of acid-suppressing medications: participants who were taking PPIs or other acid-suppressive therapies were required to undergo a washout period of 3 days, during which these medications were gradually tapered. This ensured that symptoms were not masked by prior treatments and reflected the true severity of NERD. (2) baseline symptom assessment: at the start of the run-in period, all participants underwent a thorough assessment of their symptoms using the symptom scores. This helped establish baseline levels of reflux, heartburn, and other related symptoms, ensuring that patients were suitable for randomization and could be compared effectively across the treatment groups. (3) confirmation of eligibility: throughout the run-in period, participants were monitored for compliance with the inclusion criteria, including the negative gastroscopy results and absence of any other conditions, such as functional heartburn or reflux hypersensitivity, confirmed by multichannel intraluminal impedance-pH monitoring. Only those who remained eligible after this period proceeded to the treatment phase. (4) patient education and compliance: participants were educated on the study protocol, including the importance of adhering to the treatment schedule and keeping symptom diaries. Their compliance with these instructions was monitored through regular check-ins and follow-ups to ensure the consistency of their data.

Eligibility for the study required a confirmed diagnosis of NERD with spleen deficiency and damp-heat syndrome, as diagnosed and treated at the participating institutions between July 2020 and December 2021.

For the diagnosis of NERD, patients were required to present gastroscopy reports from Grade III Class A hospitals confirming negative endoscopic findings, with the report being issued within the past year. This one-year time frame was selected based on evidence suggesting that most GERD patients (75%−97%) maintain a stable clinical phenotype over time (18), making it reasonable to assume that endoscopic findings within this period accurately reflect the patient's current condition. Additionally, multichannel intraluminal impedance-pH monitoring was conducted on those with negative endoscopic results who were off PPI therapy, to exclude conditions such as functional heartburn and reflux hypersensitivity.

Information regarding spleen deficiency and damp-heat syndrome was collected by a senior TCM practitioner, who held an associate professor rank or higher. These practitioners were rigorously trained and supervised by the principal investigator to ensure consistency and accuracy in diagnosis.

The research protocol received approval from the Ethics Committee of Xiyuan Hospital, China Academy of Chinese Medical Sciences (Approval Number: 2020XLA009-3). The study was conducted in accordance with the Helsinki Declaration, relevant Chinese regulations, and clinical trial norms. The trial is registered with the North American Clinical Trial Data Center (ClinicalTrials.gov) under the registration number NCT04324138.

The criteria for diagnosing NERD were established based on the “Consensus Opinions on Gastroesophageal Reflux Disease in China (2006·10 Sanya)” and the “Expert Consensus Opinions on Gastroesophageal Reflux Disease in China 2014” (19, 20).

Inclusion criteria for study participants included: (1) fulfillment of the diagnostic criteria for NERD; (2) age between 18 and 70 years; (3) meeting TCM diagnostic criteria for spleen deficiency with damp-heat syndrome in NERD (Supplementary Table 1); (4) provision of informed consent and willingness to undergo the specified treatment regimen.

Exclusion criteria comprised: (1) presence of active peptic ulcers, gastrointestinal bleeding, severe gastric mucosal dysplasia, or suspected gastrointestinal tumors; (2) conditions such as distal esophageal spasm, nutcracker esophagus, achalasia, or post-achalasia surgery states; (3) functional heartburn or reflux hypersensitivity; (4) other organic diseases of the digestive system (e.g., acute or chronic pancreatitis, liver cirrhosis) or systemic diseases affecting gastrointestinal motility (e.g., scleroderma, hyperthyroidism, diabetes, chronic renal insufficiency, psychiatric and neurological disorders); (5) severe diseases of major organs, including the heart, liver, and kidneys, as well as hematological diseases and tumors; (6) pregnancy and lactation; (7) history of neurological or psychiatric disorders; (8) known allergies to the medications used in the study; (9) current participation in other clinical trials or those who have participated in such trials within the last 4 weeks.

### Intervention and medication

The JQ granules and placebo used in this trial were sourced from China Resources Sanjiu Medical & Pharmaceutical Company Limited. Both the granules and placebo were processed and manufactured uniformly by the same pharmaceutical company to ensure consistency in appearance and taste, maintaining the integrity of the double-blind study design. The JQ granules consisted of 10 herbal ingredients, whose full botanical names, authorities, plant families, pharmacopeial drug names, and drug-extract ratios are as follows: *Codonopsis pilosula* (Franch.) Nannf. [Campanulaceae; Codonopsis Radix]—Drug-extract ratio: 5:1; *Atractylodes lancea* (Thunb.) DC. [Asteraceae; Atractylodis Rhizoma]—Drug-extract ratio: 5:1; *Perilla frutescens* (L.) Britton [Lamiaceae; Perillae Folium]—Drug-extract ratio: 20:1; *Eupatorium fortunei* Turcz. [Asteraceae; Eupatorii Herba]—Drug-extract ratio: 10:1; *Citrus aurantium* L. [Rutaceae; Aurantii Fructus]—Drug-extract ratio: 10:1; *Scutellaria baicalensis* Georgi [Lamiaceae; Scutellariae Radix]—Drug-extract ratio: 10:1; *Coptis chinensis* Franch. [Ranunculaceae; Coptidis Rhizoma]—Drug-extract ratio: 10:1; *Sepiella maindroni* de Rochebrune [Sepiidae; Sepiae Endoconcha (Cuttlebone)]—drug-extract ratio: 10:1; Massa medicata fermentata (Shenqu) [Traditional Fermented Product; Massa Medicata Fermentata]—Drug-extract ratio: 5:1; and *Amomum villosum* Lour. [Zingiberaceae; Amomi Fructus Rotundus]—Drug-extract ratio: 20:1. The final drug-extract ratio for the JQ granule product is approximately 7.76:1. These details are as previously described (15).

The chemical composition of the JQ granules was analyzed using two analytical fingerprinting methods. High-performance liquid chromatography was employed for the separation and quantification of key marker compounds, as outlined in Zhang et al. (15). Additionally, ultra-performance liquid chromatography-electrospray ionization-quadrupole time-of-flight mass spectrometry was utilized to identify and confirm the chemical constituents of the granules. A total of 164 compounds were tentatively identified, including 44 flavonoids, 40 terpenoids, 43 alkaloids, 4 lignans, 2 coumarins, 2 alkyne derivatives, and 16 phenolic acids (Supplementary Figure 1, Supplementary Table 2). The identification was based on accurate mass data, retention times, and MS/MS fragmentation patterns, using established databases and literature references.

The placebo contained cyclodextrin and 5% of the active ingredients from the JQ granules, in line with previous studies that have demonstrated no significant pharmacological effects from this small percentage of active components (15, 21, 22). These studies were designed to ensure that the placebo was physiologically inert.

### Drug packaging and administration method

The experimental medication, JQ granules, and the corresponding placebo, which is a placebo of the JQ granules, are identical in packaging, appearance, color, smell, and dosage form. Both are uniformly labeled as “Clinical Research Medication” to ensure blinding integrity throughout the study. The administration guideline for both the JQ granules and placebo groups is standardized: participants are instructed to take one packet of the medication three times daily, at a one-hour interval after each meal.

The treatment duration for both groups is set at 4 weeks, with a subsequent 4-week follow-up period to monitor the long-term effects and safety of the intervention. To control for potential confounding factors, participants are required to abstain from any Chinese herbal compounds, Chinese patent medicines, acid suppressants, and prokinetic drugs for a minimum of 1 week prior to their enrollment in the study.

### Randomization and masking protocol

This study uses a double-blind design. Using SAS software version 9.4 (SAS Institute Inc., Cary, NC, USA), a randomization table was generated based on the allocation numbers and randomization ratios of the participating units. The experimental and control groups were assigned random codes in a 1:1 ratio. The selected block lengths and random seed parameters were kept confidential within the blinding protocol. Randomization is performed using SAS software version 9.4 by a statistician not directly affiliated with the clinical trial institution. Participants are randomly assigned to either the experimental group or the placebo group in a 1:1 ratio, and medications are numbered accordingly based on the generated random sequence for packaging. Subjects receive their study medications sequentially according to their enrollment order. Blinding is rigorously maintained for all parties involved, including physicians, data entry personnel, data managers, statisticians, and patients. The study uses a two-tier blinding code system. The level I blinding code indicates which drug numbers correspond to drug A or drug B, while the level II blinding code specifies whether drug A or B is the experimental drug or the placebo. Both blinding codes are held by the project management unit. The level I blinding code is provided to statisticians for analysis in accordance with the statistical analysis plan, enabling them to generate the statistical report. The second unblinding occurs during the summary meeting, where unblinding personnel sign the blinding code. The blinding codes are securely stored at the National Drug Clinical Trial Base Office at Xiyuan Hospital of the China Academy of Chinese Medical Sciences.

### Primary outcome and secondary outcomes

The primary outcomes of this study are the efficacy rates in alleviating symptoms like reflux and heartburn, and the corresponding changes in Visual Analog Scale (VAS) scores for these symptoms (15).

Secondary outcomes include a variety of assessments, such as changes in atypical symptom scores, total TCM syndrome scores, the efficacy of TCM syndrome improvements, variations in Gastroesophageal Reflux Disease Health-Related Quality of Life (GERD-HRQL) scale scores (23), shifts in the Chronic Gastrointestinal Disease Patient Reported Outcomes (PRO) Scale scores (24), and alterations in depression (SDS) and anxiety (SAS) scores (25, 26).

#### Efficacy evaluation for symptom improvement

During the medication period, participants are required to document daily occurrences of reflux and heartburn within a 24-h period on a symptom diary card. They rate the severity of these symptoms using a VAS. The average VAS scores for each symptom are calculated over the 4-week treatment period and the subsequent 4-week follow-up period. Efficacy is evaluated using the Nimodipine method, as outlined in the “Guiding principle of clinical research on new drugs of traditional Chinese medicine” (27). The formula for the Efficacy Index is: Efficacy Index = (Score before treatment – Score after treatment)/Score before treatment × 100 %. The classifications for efficacy are as follows: Cured: Efficacy Index ≥ 95%; Markedly effective: Efficacy Index ≥ 70% but < 95%; Effective: Efficacy Index ≥ 30% but < 70%; and Ineffective: Efficacy Index < 30%.

An average VAS score reduction of ≥ 30% from baseline for either reflux or heartburn symptoms at both 4 and 8 weeks of treatment is defined as effective. The Symptom Improvement Efficacy Rate is calculated using the formula: Symptom Improvement Efficacy Rate = (Number of cases cured + markedly effective + effective)/Total number of patients × 100%.

#### Atypical symptoms assessment

Atypical symptoms, such as coughing and asthma, are assessed for frequency and severity (Supplementary Table 3). These are compared between the experimental and control groups at various time points, as well as within each group at baseline, after 4 weeks of treatment, and after 4 weeks of follow-up.

#### TCM syndrome score assessment

The total score of TCM syndromes was calculated as a composite of individual scores for symptoms associated with spleen deficiency and damp-heat syndrome in TCM. These symptoms include postprandial acid reflux, bloating, burning pain in the upper abdomen, chest discomfort, anorexia, and other related symptoms (Supplementary Table 4). The frequency and severity of these symptoms were assessed using a standardized scoring system, and the total score was compared between the experimental and control groups at baseline, after 4 weeks of treatment, and after 4 weeks of follow-up. Additionally, within-group comparisons were performed at each time point.

#### TCM syndrome efficacy rate

A reduction of ≥ 30% in the total TCM syndrome score from baseline at both 4 and 8 weeks of treatment is defined as effective. The TCM Syndrome Efficacy Rate is calculated using the formula: TCM Syndrome Efficacy Rate = (Number of cases cured + markedly effective + effective)/Total number of patients × 100 %.

#### Chronic Gastrointestinal Disease PRO Scale score assessment

The Chronic Gastrointestinal Disease PRO Scale is categorized into six dimensions: reflux, dyspepsia, abnormal defecation, systemic symptoms, psychological symptoms, and social function. Scores for each dimension and the total score of the Chronic Gastrointestinal Disease PRO Scale were compared between the experimental group and the control group at various time points. Comparisons were also made within each group at baseline, after 4 weeks of treatment, and at 4 weeks of follow-up.

### Safety detection indicators and adverse event monitoring

Safety detection indicators encompassed routine assessments of blood, urine, and stool, occult blood tests, liver function tests (encompassing alanine aminotransferase [ALT] and aspartate aminotransferase [AST]), renal function tests (including blood urea nitrogen [BUN] and creatinine), and electrocardiograms. Adverse events or unintended toxic side effects (encompassing symptoms, signs, and laboratory findings) were meticulously monitored and reported. Each adverse event was analyzed and evaluated, followed by systematic tracking and documentation. Incidents occurring during the study were meticulously recorded in case report forms, detailing the symptoms, severity, onset time, duration, management measures, progression, and outcome, and assessing their correlation with the study medication.

### Statistical analysis

The statistical analysis was designed based on prior research. The efficacy rate of JQ granules combined with low-dose omeprazole in treating NERD was reported to be 91% (15), while the average efficacy rate for placebo was documented as 60% (28, 29). The study aimed to conduct a superiority test for two independent proportions. Parameters for the test included a clinically significant difference (Δ) of 0.031, a significance level (α) of 0.05, and a power (β) of 0.2, with a 1:1 sample size ratio. Assuming a 20% loss to follow-up, the estimated sample size was 78 participants, with 39 in each group.

Demographic data and baseline indicators were analyzed to ensure comparability between the two groups. Compliance analysis was conducted to verify adherence to the prescribed drug regimen, documenting any deviations, including prohibited or concomitant medications.

For efficacy analysis, the primary efficacy indicators were evaluated using both per-protocol analysis and intention-to-treat analysis, with a primary focus on the full analysis set (FAS). Center effects on efficacy indicators were adjusted using the Cochran-Mantel-Haenszel (CMH) test, ensuring robust assessments. Additional statistical methods included the *t*-test, paired *t*-test, rank-sum test, paired rank-sum test, median test, Chi-square test, Fisher's exact test, and log-rank test. For confounding factors such as inter-group imbalance before treatment, the Cox proportional hazards model was applied to eliminate their influence on efficacy comparisons. Two-sided tests with a *P* ≤ 0.05 were considered statistically significant.

Safety analysis was performed using the safety analysis set (SS), providing a comprehensive evaluation of the safety profile of the trial drugs. Adverse effects were meticulously documented to ensure participant safety.

## Results

### Patient characteristics

**Figure 1:**
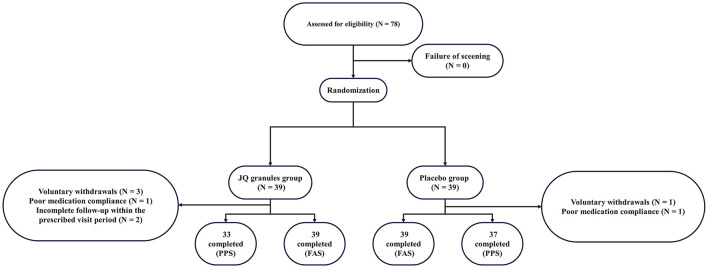
Study workflow diagram.

The first patient was enrolled on July 2, 2020, and the last patient was enrolled on December 30, 2021. A total of 78 subjects were enrolled in this study, with 39 in the placebo group and 39 in the JQ granules group, all of whom received at least one treatment and thus all entered the SS. Adhering to the intention-to-treat principle, 39 in the placebo group and 39 in the JQ granules group were included in the FAS. In the placebo group, 1 subject withdrew voluntarily, and another was non-compliant with medication; in the JQ granules group, 3 subjects withdrew voluntarily, 1 was non-compliant with medication, and 2 missed the follow-up visits within the prescribed period. Consequently, 37 in the placebo group and 33 in the JQ granules group were included in the PPS. The study workflow is presented in Figure 1.

In the placebo group, there were 18 males (46.15%) and 21 females (53.85%), with an average age of 48.49 ± 16.22 years and an average weight of 60.73 ± 9.85 kg. In the JQ granules group, there were 16 males (41.03%) and 23 females (58.97%), with an average age of 42.64 ± 14.49 years and an average weight of 63.86 ± 13.49 kg. The differences in demographic data between the two groups were not statistically significant (*P* > 0.05) (Table 1, Supplementary Table 5).

**Table 1:** Demographic characteristics of the FAS patient cohort.

**Characteristics**	**Placebo group**	**JQ granules group**	***P*-value**
**Gender**, ***n*** **(%)**^**a**^			0.6479
Male	18 (46.15)	16 (41.03)	
Female	21 (53.85)	23 (58.97)	
**Ethnicity**, ***n*** **(%)**^**b**^			1
Han	38 (97.44)	37 (94.87)	
Other	1 (2.56)	2 (5.13)	
**Marital status**, ***n*** **(%)**^**a**^			0.7226
Single	9 (23.08)	10 (25.64)	
Married, living together	27 (69.23)	26 (66.67)	
Married, not living together	2 (5.13)	2 (5.13)	
Divorced	0 (0)	1 (2.56)	
Widowed	1 (2.56)	0 (0)	
**Employment**, ***n*** **(%)**^**a**^			0.797
Mental work	26 (66.67)	26 (68.42)	
Physical work	7 (17.95)	8 (21.05)	
Other	6 (15.38)	4 (10.53)	
**Education level**, ***n*** **(%)**^**a**^			0.5877
Illiterate (< 1 year)	1 (2.63)	0 (0)	
Primary school (1–6 years)	3 (7.89)	2 (5.13)	
Junior high school (7–9 years)	8 (21.05)	8 (20.51)	
High school/vocational school (10–12 years)	7 (18.42)	4 (10.26)	
College and above (≥13 years)	19 (50)	25 (64.1)	

^a^Chi-square test.

^b^Fisher's exact test.

JQ, JianpiQinghua.

Additionally, there were no statistically significant differences between the two groups in terms of medication compliance, the number of concomitant medications used previously, the distribution of concomitant medications during the study period, past medical history, and history of allergies (*P* > 0.05) (Supplementary Table 6).

### Primary outcomes

#### Efficacy rate of improvement in reflux and heartburn symptoms

Since the PPS analysis confirmed the efficacy of JQ granules, with trends consistent with those observed in the FAS analysis, this reinforces the robustness of the findings and justifies the reliance on FAS as the primary analysis. This approach ensures adherence to the intention-to-treat principle, which is essential for minimizing bias in randomized controlled trials.

**Figure 2:**
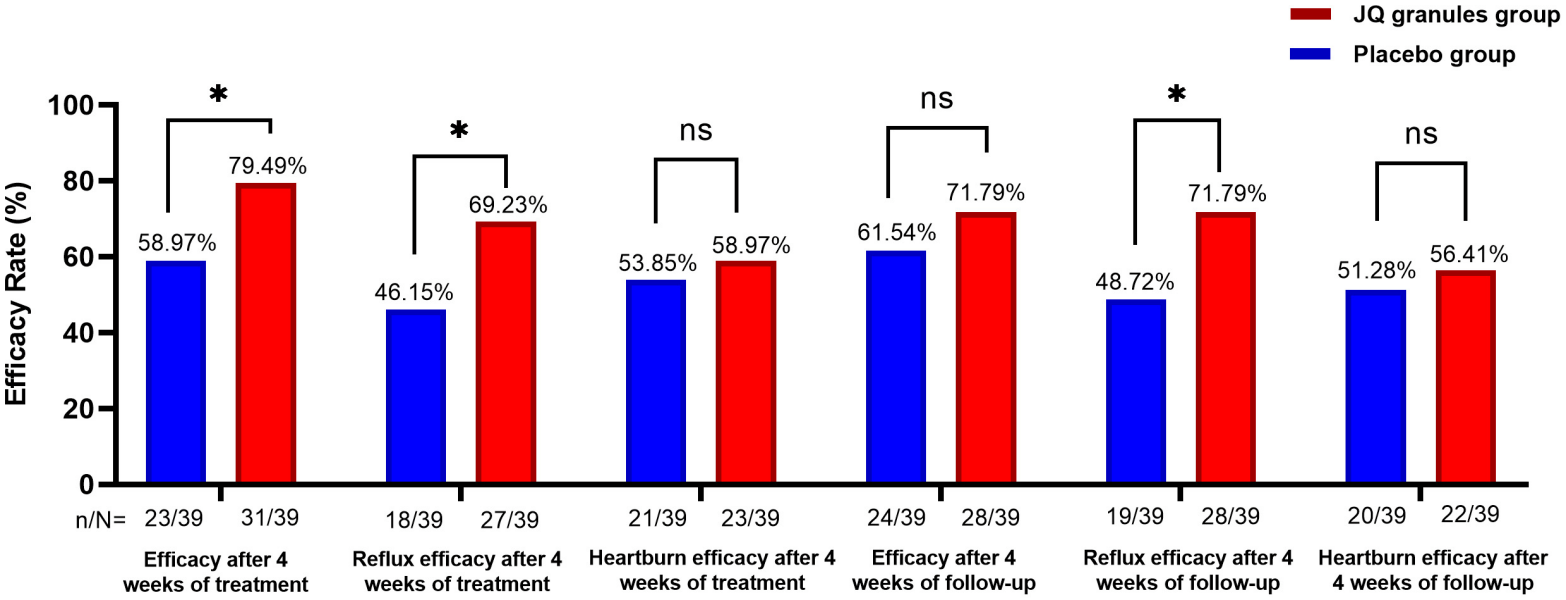
Comparison of overall efficacy rates, and efficacy rates of reflux and heartburn symptom improvement, between the JQ granules group and the placebo group over the 4-week treatment period and follow-up. The JQ granules group demonstrated a statistically significant improvement in overall efficacy rates and reflux symptoms after 4 weeks of treatment (*P < 0.05) and during the 4-week follow-up period (*P* < 0.05 for reflux symptoms). However, no significant improvement was observed in efficacy rates for heartburn symptoms after 4 weeks of treatment or during the 4-week follow-up period (ns, not significant; *P* > 0.05). Additionally, overall efficacy rates during the follow-up period were not significantly improved (ns, not significant; *P* > 0.05). Data are expressed as efficacy rates (%), with the sample size (n/N) provided below each bar. Statistical significance is indicated by *P* < 0.05.

The FAS analysis revealed a statistically significant difference in the overall efficacy rates for alleviating reflux and heartburn symptoms between the placebo and JQ granules groups after a 4-week treatment. The placebo group showed an efficacy rate of 58.97%, while the JQ granules group had a higher rate of 79.49% (*P* < 0.05) (Table 2, Figure 2). For reflux symptoms, the efficacy rate was 46.15% in the placebo group and 69.23% in the JQ granules group (*P* < 0.05), while for heartburn symptoms, the rates were 53.85% and 58.97%, respectively, with no significant difference (*P* > 0.05) (Table 2, Figure 2).

**Table 2:** Effectiveness of treatment on reflux and heartburn symptoms in the FAS patient population.

	**Placebo group**	**JQ granules group**	***P*-value**
	***n*** **(%)**	***n*** **(%)**	
n	39	39	
Efficacy after 4 weeks of treatment^**a**^	23 (58.97)	31 (79.49)	0.0478
Reflux efficacy after 4 weeks of treatment^**b**^	18 (46.15)	27 (69.23)	0.0391
Heartburn efficacy after 4 weeks of treatment^**b**^	21 (53.85)	23 (58.97)	0.6479
Efficacy after 4 weeks of follow-up^**a**^	24 (61.54)	28 (71.79)	0.3213
Reflux efficacy after 4 weeks of follow-up^**b**^	19 (48.72)	28 (71.79)	0.0373
Heartburn efficacy after 4 weeks of follow-up^**b**^	20 (51.28)	22 (56.41)	0.6496

^a^Cochran-Mantel-Haenszel test.

^b^Chi-square test.

JQ, JianpiQinghua; N, Number of participants.

However, at the 4-week follow-up, the efficacy rates were 61.54% for the placebo group and 71.79% for the JQ granules group, with no significant difference observed (*P* > 0.05), as detailed in Table 2, Figure 2. However, the efficacy rate for reflux improvement during the follow-up period was 48.72% for the placebo group and 71.79% for the JQ granules group, with a significant difference observed (*P* < 0.05). The efficacy rates for heartburn improvement were 51.28% for the placebo group and 56.41% for the JQ granules group, with no significant difference (*P* > 0.05) (Table 2, Figure 2).

#### Reflux VAS scores

**Figure 3:**
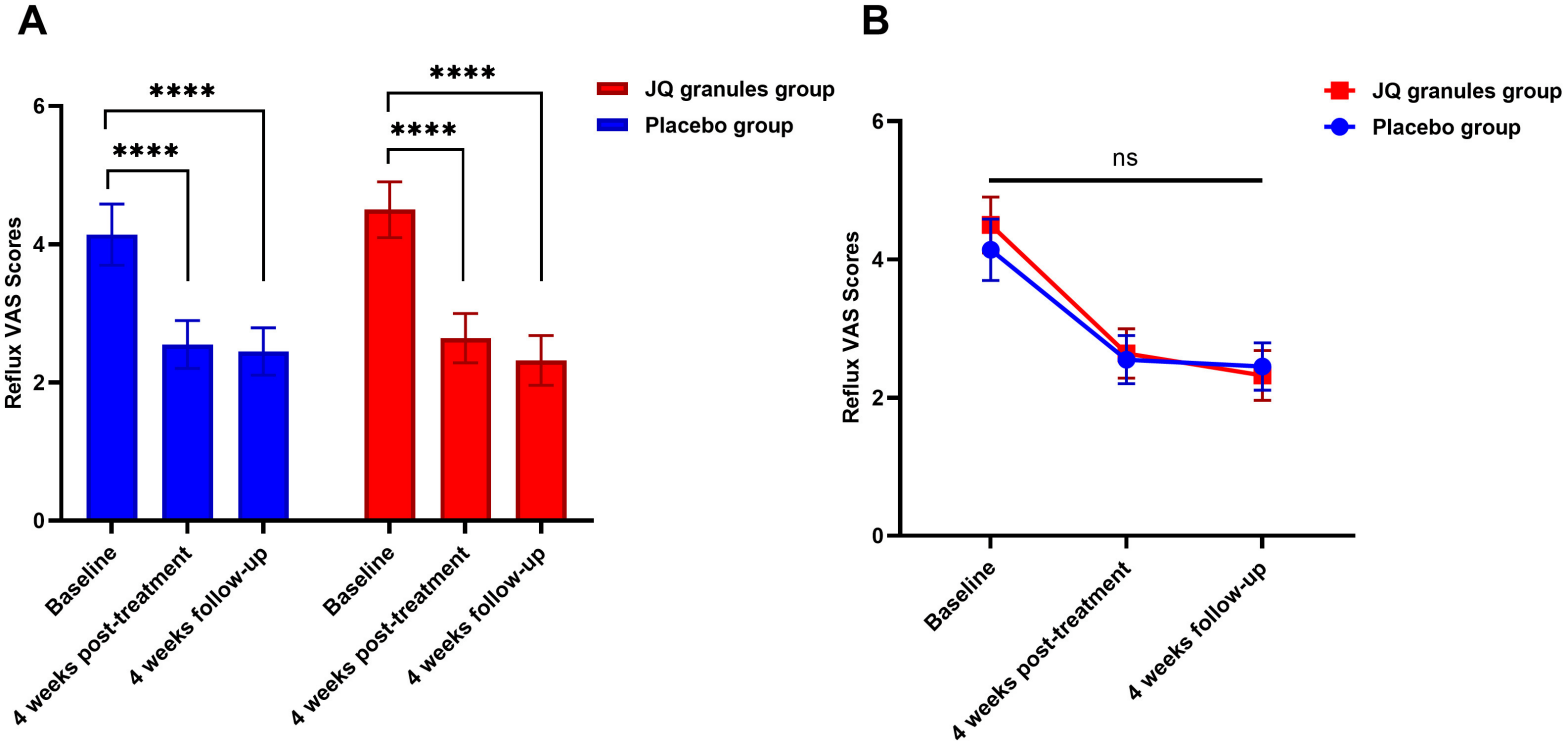
Comparison of reflux VAS scores between the JQ granules group and the placebo group. **(A)** Bar graph showing the mean reflux VAS scores at baseline, after 4 weeks of treatment, and at the 4-week follow-up for both the JQ granules group (red bars) and the placebo group (blue bars). Both groups demonstrated a significant reduction in VAS scores from baseline to the 4-week post-treatment and the 4-week follow-up (^****^P < 0.0001). **(B)** Line graph illustrating the mean reflux VAS scores at baseline, after 4 weeks of treatment, and at the 4-week follow-up for both the JQ granules group (red line) and the placebo group (blue line). No statistically significant differences were observed between the two groups at the 4-week post-treatment and the 4-week follow-up (ns, not significant; *P* > 0.05). The trend shows a consistent decrease in VAS scores over time for both groups. Statistical significance is denoted by ^****^P < 0.0001.

Within the FAS, both the JQ granules group and the placebo group showed a significant reduction in reflux VAS scores from baseline after 4 weeks of treatment and at the subsequent 4-week follow-up (*P* < 0.0001 for both groups). This reduction is highlighted in Table 3 and depicted in Figure 3A.

**Table 3:** Evaluation of reflux VAS scores in the FAS patient group.

	**Placebo group**	**JQ granules group**	***P*-value**
**Baseline** ^**a**^			0.5469
N (Nmiss)	39 (0)	39 (0)	
Mean (SD)	4.14 ± 2.77	4.5 ± 2.52	
**4 weeks post-treatment** ^**a**^			0.8537
N (Nmiss)	38 (1)	37 (2)	
Mean (SD)	2.55 ± 2.14	2.64 ± 2.17	
**4 weeks follow-up** ^**a**^			0.7992
N (Nmiss)	38 (1)	37 (2)	
Mean (SD)	2.45 ± 2.11	2.32 ± 2.19	
**4 weeks of treatment vs. baseline** ^**b**^			0.4257
N (Nmiss)	38 (1)	37 (2)	
Mean (SD)	−1.5 ± 1.78	−1.97 ± 1.7	
*P*-value^c^	< 0.0001	< 0.0001	
**4 weeks of follow-up vs. baseline** ^**b**^			0.1934
N (Nmiss)	38 (1)	37 (2)	
Mean (SD)	−1.6 ± 1.77	−2.29 ± 1.94	
*P*-value^c^	< 0.0001	< 0.0001	

^a^t-test.

^b^Analysis of covariance.

^c^Paired t-test within group.

JQ, JianpiQinghua; N, Number of participants; Nmiss, Number of missing values; SD, Standard Deviation.

However, no statistically significant differences were found in VAS scores between the JQ granules and placebo groups at either the 4-week treatment or the 4-week follow-up (*P* > 0.05), as indicated in Figure 3B. Figure 3B consistently demonstrates a downward trend in VAS scores for both groups over all the observed time points.

#### Heartburn VAS scores

**Figure 4:**
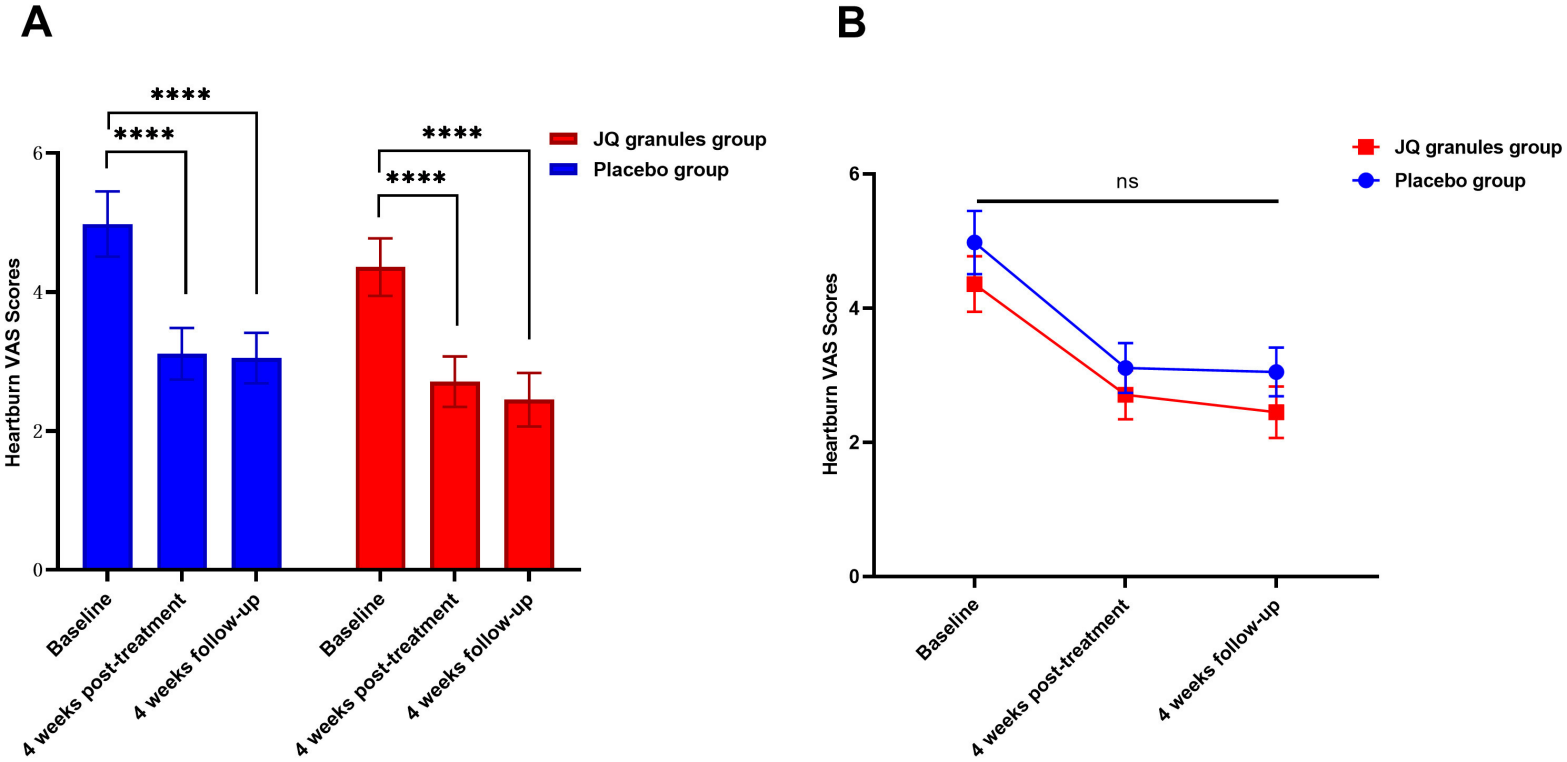
Comparison of heartburn VAS scores between the JQ granules group and the placebo group. **(A)** Bar graph showing the mean heartburn VAS scores at baseline, after 4 weeks of treatment, and at the 4-week follow-up for both the JQ granules group (red bars) and the placebo group (blue bars). Both groups demonstrated a significant reduction in VAS scores from baseline to the 4-week post-treatment and the 4-week follow-up (^****^P < 0.0001). **(B)** Line graph illustrating the mean heartburn VAS scores at baseline, after 4 weeks of treatment, and at the 4-week follow-up for both the JQ granules group (red line) and the placebo group (blue line). No statistically significant differences were observed between the two groups at the 4-week post-treatment and the 4-week follow-up (ns, not significant; *P* > 0.05). The trend shows a consistent decrease in VAS scores over time for both groups. Statistical significance is denoted by ^****^P < 0.0001.

Within the FAS, both the JQ granules group and the placebo group experienced a significant decrease in heartburn VAS scores at the conclusion of the 4-week treatment and at the 4-week follow-up, as compared to their baseline scores (*P* < 0.0001 for both groups). The data is presented in Table 4 and graphically represented in Figure 4A. Despite these improvements, there was no statistically significant difference in VAS scores between the two groups at either the end of treatment or the follow-up stage (*P* > 0.05), as noted in Figure 4B. Figure 4B confirms a consistent downward trend in heartburn VAS scores for both groups throughout the study period.

**Table 4:** Evaluation of heartburn VAS scores in the FAS patient group.

	**Placebo group**	**JQ granules group**	***P*-value**
**Baseline** ^**a**^			0.3287
N (Nmiss)	39 (0)	39 (0)	
Mean (SD)	4.98 ± 2.94	4.36 ± 2.58	
**4 weeks post-treatment** ^**a**^			0.4459
N (Nmiss)	38 (1)	37 (2)	
Mean (SD)	3.11 ± 2.29	2.71 ± 2.21	
**4 weeks follow-up** ^**a**^			0.2611
N (Nmiss)	38 (1)	37 (2)	
Mean (SD)	3.05 ± 2.24	2.45 ± 2.34	
**4 weeks of treatment vs. baseline** ^**b**^			0.4825
N (Nmiss)	38 (1)	37 (2)	
Mean (SD)	−1.79 ± 1.64	−1.75 ± 1.71	
*P*-value^c^	< 0.0001	< 0.0001	
**4 weeks of follow-up vs. baseline** ^**b**^			0.2384
N (Nmiss)	38 (1)	37 (2)	
Mean (SD)	−1.84 ± 1.62	−2 ± 1.9	
*P*-value^c^	< 0.0001	< 0.0001	

^a^t-test.

^b^Analysis of Covariance.

^c^Paired t-test within group.

JQ, JianpiQinghua; N,: Number of participants; Nmiss, Number of missing values; SD, Standard Deviation.

### Secondary outcomes

#### Atypical symptom scores

In the placebo group, there was a non-significant reduction in the frequency and severity of cough post-treatment (*P* > 0.05). Conversely, the JQ granules group experienced a significant decrease in cough-related scores (*P* < 0.01). Both groups showed a significant reduction in cough scores at the four-week follow-up compared to baseline (*P* < 0.05 for both groups), as documented in Supplementary Table 7. Nevertheless, no significant differences in cough scores were noted between the groups at either time point (*P* > 0.05), according to Supplementary Table 7.

Regarding asthma frequency, the placebo group showed a non-significant increase after treatment and at the follow-up (*P* > 0.05), while the JQ granules group had a non-significant decrease in scores (*P* > 0.05) (Supplementary Table 8). No significant differences in asthma frequency scores were observed between the groups at the treatment or follow-up stages (*P* > 0.05), as per Supplementary Table 8. Similarly, both groups experienced non-significant decreases in asthma severity scores post-treatment and at the follow-up (*P* > 0.05) (Supplementary Table 8). There were no significant differences in asthma severity scores between the groups at either the treatment or follow-up stages (*P* > 0.05), as detailed in Supplementary Table 8.

**Figure 5:**
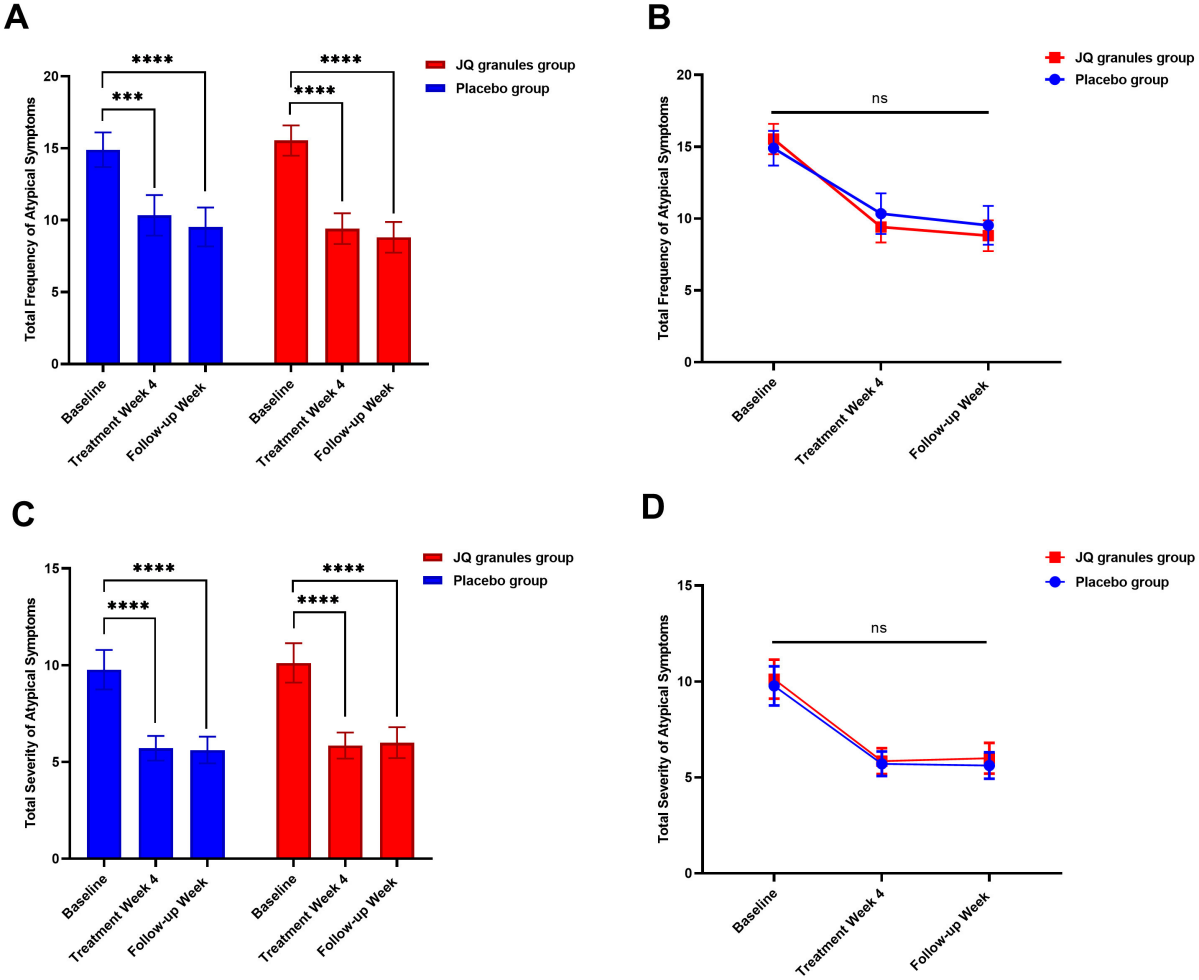
Comparison of the total frequency and severity of atypical symptoms between the JQ granules group and the placebo group. **(A)** Bar graph showing the total frequency of atypical symptoms at baseline, after 4 weeks of treatment, and at the follow-up for both the JQ granules group (red bars) and the placebo group (blue bars). Both groups showed a significant reduction in symptom frequency from baseline to the 4-week post-treatment and the follow-up (^***^P < 0.001; ^****^P < 0.0001). **(B)** Line graph illustrating the total frequency of atypical symptoms at baseline, after 4 weeks of treatment, and at the follow-up for both the JQ granules group (red line) and the placebo group (blue line). No statistically significant differences were observed between the two groups at any time point (ns, not significant; *P* > 0.05). A trend indicates that the JQ granules group had a more pronounced improvement in symptom frequency. **(C)** Bar graph showing the total severity of atypical symptoms at baseline, after 4 weeks of treatment, and at the follow-up for both the JQ granules group (red bars) and the placebo group (blue bars). Both groups showed a significant reduction in symptom severity from baseline to the 4-week post-treatment and the follow-up (^****^P < 0.0001). **(D)** Line graph illustrating the total severity of atypical symptoms at baseline, after 4 weeks of treatment, and at the follow-up for both the JQ granules group (red line) and the placebo group (blue line). No statistically significant differences were observed between the two groups at any time point (ns, not significant; *P* > 0.05). Statistical significance is denoted by ^**^P < 0.001 and ^****^P < 0.0001.

Both the JQ granules group and the placebo group reported a significant reduction in the total frequency and severity of atypical symptoms after 4 weeks of treatment and at the follow-up, as compared to their baseline scores (*P* < 0.05 for both groups) (Table 5, Figures 5A, C). Throughout the study, no statistically significant differences were found between the two groups at any of the visits (P > 0.05) (Figures 5B, D). However, there was a noticeable trend indicating that the JQ granules group had a more pronounced improvement in the total score of atypical symptom frequency relative to the placebo group, both at the 4-week treatment and during the follow-up period, as illustrated in Figure 5B.

**Table 5:** Analysis of total frequency and severity of atypical symptom scores.

	**Placebo group**	**JQ granules group**	***P*-value**
**Total frequency of atypical symptoms**			
**Baseline** ^**a**^	14.9 ± 7.52	15.54 ± 6.58	0.63782
N (Nmiss)	39 (0)	39 (0)	
**Treatment week 4** ^**a**^	10.34 ± 8.71	9.41 ± 6.5	0.89425
N (Nmiss)	38 (1)	37 (2)	
**Follow-up week 4** ^**a**^	9.53 ± 8.32	8.81 ± 6.51	0.89844
N (Nmiss)	38 (1)	37 (2)	
**4 weeks of treatment vs. baseline** ^**b**^	−4.74 ± 6.86	−5.84 ± 6.37	0.2848
N (Nmiss)	38 (1)	37 (2)	
*P*-value^c^	0.00011	0	
**4 weeks of follow-up vs. baseline** ^**b**^	−5.55 ± 7.26	−6.43 ± 5.69	0.4184
N (Nmiss)	38 (1)	37 (2)	
*P*-value^c^	0.00001	0	
**Total severity of atypical symptoms**			
**Baseline** ^**a**^	9.77 ± 6.38	10.12 ± 6.34	0.68486
N (Nmiss)	39 (0)	39 (0)	
**Treatment week 4** ^**a**^	5.71 ± 3.92	5.85 ± 4.11	0.91948
N (Nmiss)	38 (1)	37 (2)	
**Follow-up week 4** ^**a**^	5.62 ± 4.26	6 ± 4.85	0.88151
N (Nmiss)	38 (1)	37 (2)	
**4 weeks of treatment vs. baseline** ^**b**^	−4.18 ± 5.4	−4.33 ± 5.59	0.738
N (Nmiss)	38 (1)	37 (2)	
*P*-value^c^	0	0	
**4 weeks of follow-up vs. baseline** ^**b**^	−4.28 ± 5.35	−4.18 ± 5.61	0.9507
N (Nmiss)	38 (1)	37 (2)	
*P*-value^c^	0	0	

^**a**^Rank-sum test.

^b^Analysis of covariance.

^c^Paired rank-sum test within group.

JQ, JianpiQinghua; N, Number of participants; Nmiss, Number of missing values.

#### Total score of TCM syndromes

In the FAS analysis, both the JQ granules and placebo groups demonstrated a statistically significant decrease in the total score of TCM syndromes after 4 weeks of treatment and at the 4-week follow-up (*P* < 0.0001) (Table 6). However, no significant differences were observed between the two groups at either the 4-week treatment or follow-up timepoints (*P* > 0.05) (Table 6). Both groups showed a consistent downward trend in TCM syndrome scores throughout the study. While the JQ granules group exhibited a trend toward better improvement in total TCM syndrome scores compared to the placebo group after 4 weeks of treatment, this difference was not statistically significant.

**Table 6:** Analysis of total score of TCM syndromes.

		**Placebo group**	**JQ granules group**	***P*-value**
**Total TCM syndrome scores**				
Baseline^a^	N (Nmiss)	39 (0)	39 (0)	
	Median (Q1, Q3)	10 (7, 14)	11 (6, 14)	0.79058
	Min, Max	1, 22	2, 19	
Treatment week 4^a^	N (Nmiss)	38 (1)	37 (2)	
	Median (Q1, Q3)	6 (3, 10)	5 (3, 9)	0.15375
	Min, max	0, 17	0, 16	
	N (Nmiss)	38 (1)	37 (2)	
Follow-up week 4^a^	Median (Q1, Q3)	6 (3, 10)	5 (3, 9)	0.63143
	Min, max	0, 15	0, 17	
4 weeks of treatment vs. baseline^b^	N (Nmiss)	38 (1)	37 (2)	
	Median (Q1, Q3)	−3 (−6, 0)	−4 (−8, −2)	0.0781
	Min, Max	−11, 4	−14, 5	
	*P*-value^c^	< 0.0001	< 0.0001	
4 weeks of follow-up vs. baseline^b^	N (Nmiss)	38 (1)	37 (2)	
	Median (Q1, Q3)	−3 (−5, −2)	−4 (−6, −2)	0.3501
	Min, Max	−13, 10	−14, 4	
	*P*-value^c^	< 0.0001	< 0.0001	

^a^Rank-sum test.

^b^Analysis of covariance.

^c^Paired rank-sum test within group.

JQ, JianpiQinghua; N, Number of participants; Nmiss, Number of missing values.

Quartile 1: Q1; Quartile 3: Q3; Minimum value: Min; Maximum value: Max.

#### Efficacy rate of improvement in TCM syndromes

After a 4-week treatment period, the JQ granules group exhibited a higher efficacy rate in improving TCM syndromes, with 69.23%, compared to the placebo group, which had an efficacy rate of 46.15%. This difference was statistically significant (*P* < 0.05). However, at the 4-week follow-up, the efficacy rates were 56.41% for the placebo group and 53.85% for the JQ granules group, with no statistically significant difference between the two groups (*P* > 0.05) (Table 7).

**Table 7:** Efficacy rate of improvement in TCM syndromes.

	**Placebo group**	**JQ granules group**	***P*-value**
*N* (%)	39	39	
Efficacy after 4 weeks of treatment^**a**^	18 (46.15)	27 (69.23)	0.0254
Efficacy after 4 weeks of follow-up^**a**^	22 (56.41)	21 (53.85)	0.8978

^a^Cochran-Mantel-Haenszel test.

JQ, JianpiQinghua; N, Number of participants.

#### Analysis of GERD-HRQL scale score variations

After the 4-week treatment and subsequent follow-up, both the JQ granules and the placebo groups exhibited significant reductions in GERD-HRQL scores from the baseline levels (P < 0.01 for JQ granules, P < 0.001 for the placebo group) (Supplementary Figure 2A). However, there were no significant differences between the two groups in terms of score changes post-treatment or at the follow-up (P > 0.05), as detailed in Supplementary Table 9 and depicted in Supplementary Figure 2B. Supplementary Figure 2B shows a consistent downward trend in scores for both groups, with a non-significant trend suggesting a slight advantage for the JQ granules group at the 4-week follow-up.

#### Shifts in the Chronic Gastrointestinal Disease PRO scale scores

After a 4-week treatment period and at the subsequent 4-week follow-up, both the JQ granules and placebo groups exhibited significant reductions in scores for dyspepsia, bowel irregularity, and psychological mood compared to baseline (*P* < 0.0001 for both groups). These findings are detailed in Supplementary Table 10 and illustrated in Supplementary Figures 3A–C. No significant differences were observed between the groups at the post-treatment or follow-up assessments (P > 0.05), as shown in Supplementary Figures 4A–C. Supplementary Figures 4A–C consistently displays a downward trend in scores for both groups across all measured time points, with a non-significant trend suggesting that the JQ granules group may have had marginally better outcomes than the placebo group in bowel irregularity scores.

Following the 4-week treatment, the JQ granules and placebo groups both showed significant reductions in reflux dimension scores relative to baseline (*P* < 0.001 for JQ granules, *P* < 0.05 for placebo), as documented in Supplementary Table 10, Supplementary Figure 3D. At the 4-week follow-up, the placebo group did not exhibit significant changes from the post-treatment scores (*P* > 0.05), while the JQ granules group maintained a significant decrease from baseline (*P* < 0.01). Despite these intragroup changes, no significant differences in reflux dimension scores were found between the JQ granules and placebo groups at either the 4-week post-treatment or the 4-week follow-up (*P* > 0.05) (Supplementary Figure 4D). Supplementary Figure 4D indicates a consistent downward trend in scores for both groups, with a non-significant trend suggesting the JQ granules group may have performed slightly better.

At the conclusion of the 4-week treatment period, the placebo group exhibited a significant reduction in systemic symptom dimension scores when compared to their pre-treatment levels (*P* < 0.05), as documented in Supplementary Table 10 and illustrated in Supplementary Figure 3E. In contrast, the JQ granules group did not demonstrate a statistically significant decrease at this stage (*P* > 0.05). By the 4-week follow-up, however, both the JQ granules and placebo groups had achieved significant reductions in systemic symptom dimension scores relative to their pre-treatment values (JQ granules group: *P* < 0.05; placebo group: *P* < 0.001). Despite these intragroup improvements, no significant differences were observed between the two groups in terms of systemic symptom dimension scores at either the 4-week post-treatment or the 4-week follow-up assessments (*P* > 0.05), as shown in Supplementary Figure 4E. Supplementary Figure 4E visually represents the consistent downward trend in systemic symptom dimension scores for both groups across all measured time points, underscoring the overall improvement in systemic symptoms experienced by participants in both the JQ granules and placebo groups.

After 4 weeks of treatment, the placebo group showed a decrease in social functioning dimension scores, but this was not statistically significant (*P* > 0.05). In contrast, the JQ granules group exhibited a statistically significant reduction (*P* < 0.01). At the 4-week follow-up, both groups had significant decreases in scores from pre-treatment (*P* < 0.01 for both). The JQ granules group had lower scores than the placebo group at both the 4-week post-treatment and the 4-week follow-up, which was statistically significant (*P* < 0.05). Supplementary Table 10, Supplementary Figure 3F document these findings, with a line graph (Supplementary Figure 4F) showing a downward trend in social functioning dimension scores for both groups.

Both the JQ granules and placebo groups showed a statistically significant decrease in the total scores of the Chronic Gastrointestinal Disease PRO Scale following the 4-week treatment and at the 4-week follow-up when compared to baseline scores (*P* < 0.0001 for both groups). No statistically significant differences were observed between the JQ granules and placebo groups at the 4-week post-treatment or the 4-week follow-up (*P* > 0.05). Supplementary Table 10, Supplementary Figure 3G present these results, with a line graph (Supplementary Figure 4G) indicating a consistent downward trend in total PRO Scale scores for both groups throughout the study period. Additionally, a non-significant trend suggests that the JQ granules group may have shown a better improvement in total PRO Scale scores compared to the placebo group at both the 4-week treatment and follow-up (Supplementary Figure 4G).

### Alterations in SDS and SAS scores

**Figure 6:**
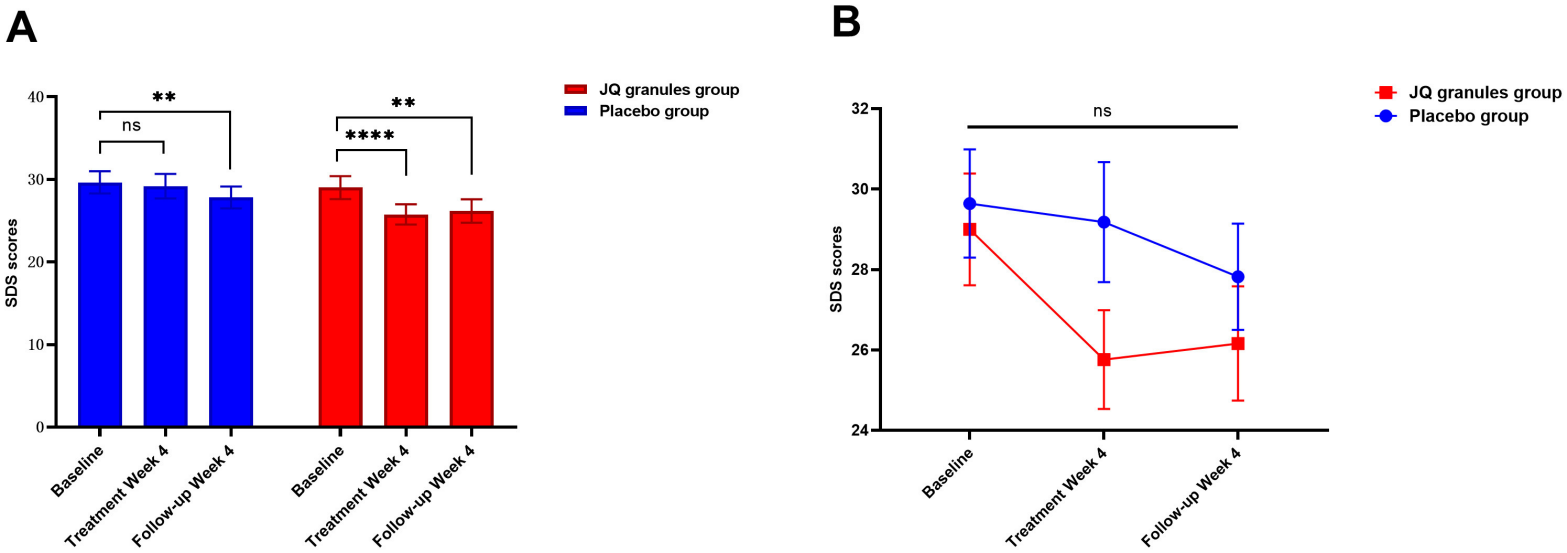
Reduction in SDS scores for the placebo (blue bars) and JQ granules groups (red bars) over the 4-week treatment period and follow-up. **(A)** The placebo group exhibited a reduction in SDS scores, which was not statistically significant (ns, not significant; *P* > 0.05) over the 4-week treatment period. In contrast, the JQ granules group showed a statistically significant reduction in SDS scores (^****^P < 0.0001). Both groups displayed a significant decrease in scores at the 4-week follow-up compared to their baseline assessments (^**^P < 0.01 for both). **(B)** Comparison of SDS scores between the placebo (blue line) and JQ granules groups (red line) at the end of the 4-week treatment and follow-up. At the end of the treatment period, the scores of the JQ granules group were marginally lower than those of the placebo group, nearing statistical significance (*P* = 0.0607). By the 4-week follow-up, no statistically significant difference in scores was observed between the JQ granules and placebo groups (ns, not significant; *P* > 0.05). Both groups demonstrated a consistent downward trend in SDS scores across all measured time points, with the JQ granules group possibly showing a greater improvement trend than the placebo group at both the 4-week treatment and follow-up. Statistical significance is denoted by ^**^P < 0.01 and ^****^P < 0.0001.

After a 4-week treatment period, the placebo group exhibited a reduction in SDS scores, yet this decrease was not statistically significant (*P* > 0.05). Conversely, the JQ granules group demonstrated a statistically significant reduction in SDS scores during the same timeframe (*P* < 0.0001). A statistically significant decrease in scores was noted for both groups at the 4-week follow-up, as compared to their initial assessments (*P* < 0.01 for both) (Table 8, Figure 6A). At the end of the 4-week treatment, the scores of the JQ granules group were marginally lower than those of the placebo group, nearing statistical significance (*P* = 0.0607) (Figure 6B). By the 4-week follow-up, no statistically significant difference in scores was observed between the JQ granules and placebo groups (*P* > 0.05), as shown in Figure 6B. Figure 6B depicts a consistent downward trend in SDS scores for both groups across all measured time points, with the JQ group possibly showing a greater improvement trend than the placebo group at both the 4-week treatment and follow-up.

**Table 8:** Analysis of SDS and SAS scores.

	**Placebo group**	**JQ granules group**	***P*-value**
**SDS scores**			
**Baseline** ^**a**^	29.64 ± 8.4	29 ± 8.68	0.62675
N (Nmiss)	39 (0)	39 (0)	
**Treatment week 4** ^**a**^	29.18 ± 9.2	25.76 ± 7.47	0.0607
N (Nmiss)	38 (1)	37(2)	
**Follow-up week 4** ^**a**^	27.82 ± 8.15	26.16 ± 8.66	0.24783
N (Nmiss)	38 (1)	37 (2)	
**4 weeks of treatment vs. baseline** ^**b**^	−0.68 ± 7.61	−2.95 ± 3.89	0.1005
N (Nmiss)	38 (1)	37 (2)	
*P*-value^c^	0.23154	< 0.0001	
**4 weeks of follow-up vs. baseline** ^**b**^	−2.05 ± 5.79	−2.54 ± 4.91	0.6783
N (Nmiss)	38 (1)	37 (2)	
*P*-value^c^	0.00984	0.00188	
**SAS scores**			
**Baseline** ^**a**^	29.28 ± 7.23	29.26 ± 7.37	0.996
N (Nmiss)	39 (0)	39 (0)	
**Treatment week 4** ^**a**^	27.55 ± 7.12	27.16 ± 7.11	0.90232
N (Nmiss)	38 (1)	37 (2)	
**Follow-up week 4** ^**a**^	26.95 ± 7.16	26.57 ± 7.4	0.79751
N (Nmiss)	38 (1)	37 (2)	
**4 weeks of treatment vs. baseline** ^**b**^	−1.92 ± 5.09	−1.84 ± 4.54	0.8803
N (Nmiss)	38 (1)	37 (2)	
*P*-value^c^	0.032406	0.01921	
**4 weeks of follow-up vs. baseline** ^**b**^	−2.53 ± 4.8	−2.43 ± 4.6	0.9059
N (Nmiss)	38 (1)	37 (2)	
*P*-value^c^	0.001747	0.002909	

^a^Rank-sum test.

^b^Analysis of covariance.

^c^Paired rank-sum test within group.

**Figure 7:**
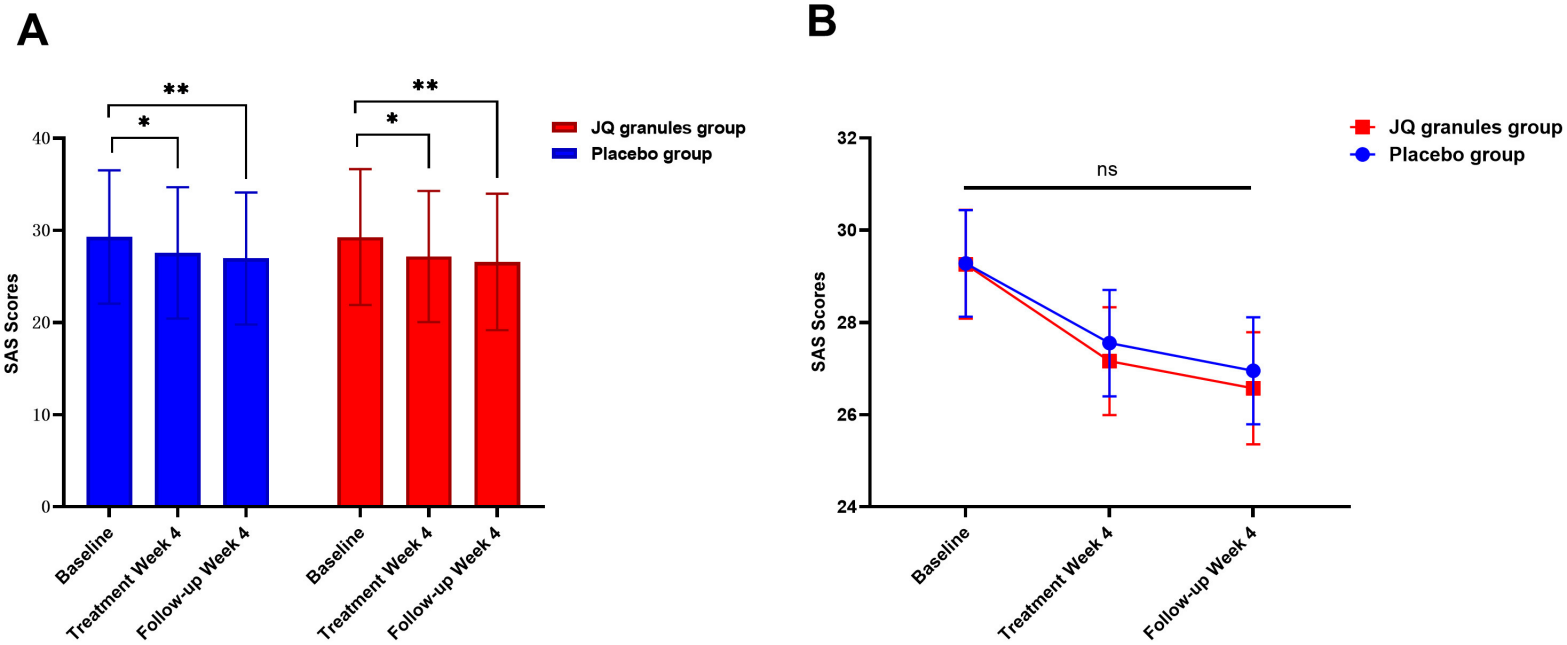
Changes in SAS scores after a 4-week treatment period and follow-up. **(A)** Reduction in SAS scores for the placebo (blue bars) and JQ granules groups (red bars) over the 4-week treatment period and follow-up. The placebo and JQ granules groups exhibited a reduction in SAS scores, which was statistically significant at both the 4-week treatment and follow-up (^*^P < 0.05; ^**^P < 0.01). Both groups displayed a significant decrease in scores compared to their baseline assessments. **(B)** Comparison of SAS scores between the placebo and JQ granules groups at the end of the 4-week treatment and follow-up. No statistically significant differences in SAS scores were observed between the two groups at either the 4-week treatment or follow-up (ns, not significant; *P* > 0.05). Both groups demonstrated a consistent decline in SAS scores across all measured time points, with a non-significant trend suggesting a potential advantage in the JQ granules group at the 4-week treatment and follow-up. Statistical significance is denoted by ^*^P < 0.05 and ^**^P < 0.01.

Similarly, both the JQ granules and placebo groups experienced a significant reduction in SAS scores after 4 weeks of treatment and at the 4-week follow-up, relative to baseline (*P* < 0.05 for both), as indicated in Table 8 and shown in Figure 7A. No statistically significant differences in SAS scores were observed between the two groups at either the 4-week treatment or follow-up (*P* > 0.05) (Figure 7B). Figure 7B indicates a consistent decline in SAS scores for both groups, with a non-significant trend suggesting a potential advantage in the JQ granules group at the 4-week treatment and follow-up.

### Safety analysis

During the trial, no statistically significant differences were observed in the incidence of adverse events between the two groups (*P* > 0.05), as documented in Table 9. The JQ granules group reported a single adverse event, which recurred twice, presenting as oral blisters and an increase in phlegm production. This event was deemed a suspected adverse reaction, potentially related to the study medication. In contrast, the placebo group reported no adverse events. Comprehensive details regarding adverse events and reactions are provided in Supplementary Table 11. Both groups exhibited no abnormalities in vital signs, including heart rate, respiration, and blood pressure. Furthermore, no serious adverse events or complications were noted in either group.

**Table 9:** Analysis of adverse event incidence.

	**Placebo group**	**JQ granules group**	***P*-value**
	***n*** **(%)**	***n*** **(%)**	
*n*	39	39	
Adverse events^a^	0 (0)	1 (2.56)	1
Adverse reactions^a^	0 (0)	1 (2.56)	1

^a^Fisher's Exact test.

## Discussion

### Overview of GERD and the role of JQ granules in NERD management

GERD is a common upper gastrointestinal disorder with significant global prevalence and notable geographical variations, affecting ~13% of individuals with weekly symptoms (30). It imposes a substantial burden on healthcare systems and negatively impacts patients' quality of life. Despite its increasing prevalence, the clinical characteristics and natural history of GERD remain incompletely understood.

Historically, GERD has been classified into three subtypes: NERD, RE, and Barrett's Esophagus. This classification follows an older progressive disease model, which has been challenged by recent studies suggesting GERD encompasses a spectrum of disorders associated with gastroesophageal junction dysfunction (31, 32). Among GERD subtypes, NERD is the most common, accounting for ~70% of patients with reflux symptoms but no visible mucosal breaks on endoscopy (33). NERD exhibits significant heterogeneity in pathophysiological mechanisms, including acid exposure disorders, reflux hypersensitivity, and functional heartburn, with the latter two considered functional esophageal disorders. This classification is debated, as reflux hypersensitivity patients often present with microscopic esophagitis and reduced chemical clearance capacity, correlating strongly with non-acid reflux and symptom production. These patients also respond well to antireflux surgery, fitting the GERD definition (34). Some scholars advocate reclassifying reflux hypersensitivity within the GERD spectrum (34).

NERD management is complicated by the limitations of acid suppression therapy. While effective for patients with abnormal esophageal acid exposure, approximately 50% of NERD patients are refractory to such treatment, and symptom recurrence is common after discontinuation (5, 35). Long-term use of PPIs is associated with potential adverse effects, such as enteric infections, osteoporosis-related fractures, and chronic kidney disease, necessitating caution in maintenance therapy (4, 36–38). Although on-demand PPI therapy is proposed as an alternative, its effectiveness in NERD patients is based on low-level evidence, as highlighted in meta-analyses (37, 38).

TCM offers distinct advantages in treating NERD. Meta-analyses indicate that pure Chinese herbal medicine is more effective than single PPI or prokinetic drug treatments, with a lower recurrence rate compared to Western medicine (39). TCM classifies NERD as “regurgitation of acid” and “esophageal inflammation,” attributing the condition to a loss of stomach harmony and the upward reversal of stomach qi. Rooted in the principles of maintaining stomach harmony and reversing stomach qi, TCM emphasizes “descending and clearing” strategies.

This approach has led to the development of JQ granules for GERD characterized by spleen deficiency and damp-heat (15). The formula aims to strengthen the spleen, clear heat, and resolve dampness, targeting the condition's underlying pathophysiological mechanisms. Preliminary clinical trials show that JQ granules, combined with a low dose of omeprazole (10 mg), are more effective in relieving NERD symptoms than the standard dose (20 mg) (15). However, high-level evidence from randomized, double-blind, placebo-controlled studies for NERD is scarce. Most GERD research focuses on RE patients and faces limitations such as small sample sizes and single-center designs.

No prospective, multicenter, randomized, double-blind, placebo-controlled intervention trials have guided the treatment of NERD with the method of strengthening the spleen and clearing heat and dampness. This study aims to address these gaps by conducting a multicenter, randomized, placebo-controlled clinical trial to evaluate the efficacy and safety of JQ granules in treating NERD. This novel approach seeks to provide robust evidence supporting the integration of TCM into NERD management.

### Demographic and sociological characteristics of the study population

This study enrolled 78 participants, including 34 males and 44 females, yielding a gender-specific incidence ratio of 0.77:1. The predominance of NERD among females is consistent with prior research, which attributes the gender difference to biological and physiological variations. Male esophageal mucosa is reportedly more susceptible to duodenogastric reflux injury, often leading to RE, whereas females are more prone to gastroesophageal reflux symptoms characteristic of NERD (40–42).

Educational level emerged as a significant factor, with higher education identified as an independent risk factor for NERD (OR = 1.66, 95% CI 1.06–2.59) (43, 44). In this cohort, the ratio of participants with a college degree or above to those with lower educational levels was 1.33:1, consistent with prior research linking higher education to increased GERD prevalence.

The age distribution of participants was also consistent with previous reports. The mean ages in the JQ granules and placebo groups were 42.64 ± 14.49 years and 48.49 ± 16.22 years, respectively. GERD prevalence in China peaks between the ages of 40–49 and 50–59, with a decline observed in individuals over 60 (45). This pattern may reflect increased work pressure and unhealthy lifestyle habits among individuals in the over-40 age group, who represent the primary workforce.

### Efficacy of JQ granules in alleviating reflux and heartburn symptoms

In this study, the primary efficacy evaluation indicators revealed significant improvements in reflux and heartburn VAS scores among patients in the JQ granules group. The FAS analysis consistently demonstrated that JQ granules effectively ameliorated reflux and heartburn symptoms in NERD patients. At the 4-week treatment period, the JQ granules group showed a significantly higher efficacy rate (79.49%) than the placebo group (58.97%) (*P* < 0.05), confirming the positive therapeutic effect of JQ granules.

The efficacy rate for reflux symptoms after 4 weeks of treatment was 46.15% for the placebo group and 69.23% for the JQ granules group, with a statistically significant difference observed between the two groups (*P* = 0.0391). However, for heartburn efficacy, no significant difference was observed between the groups (53.85% for the placebo group and 58.97% for the JQ granules group, *P* = 0.6479), suggesting that JQ granules had a similar effect to placebo on heartburn symptoms.

At the 4-week follow-up, the overall efficacy rates were 61.54% for the placebo group and 71.79% for the JQ granules group, with no statistically significant difference (*P* = 0.3213). However, JQ granules showed a significantly higher efficacy rate for reflux improvement (71.79%) compared to the placebo group (48.72%) (*P* = 0.0373). This finding highlights that JQ granules can sustain therapeutic effects on reflux symptoms even after the treatment period. On the other hand, heartburn efficacy at the 4-week follow-up showed no significant difference between the two groups (56.41% for the JQ granules group vs. 51.28% for the placebo group, *P* = 0.6496), reinforcing the conclusion that JQ granules had no distinct advantage over placebo for heartburn relief during the follow-up.

The placebo response rate of 58.97% observed in this study aligns with findings from previous NERD trials. A meta-analysis of GERD trials reported placebo response rates ranging from 2.94% to 47.06%, with higher rates commonly observed in NERD due to its functional nature (46). The response rates for placebo in controlling regurgitation symptoms ranged from 20% to 60%, depending on the trial and the definition of a successful outcome used in each study (47). Similarly, a placebo-controlled trial of vonoprazan in NERD reported a heartburn-free day proportion of 61.50% in the placebo group (48), closely comparable to the placebo response rate in this study. These findings emphasize the substantial placebo response characteristic of NERD, where symptom relief is heavily reliant on subjective evaluations.

Several factors likely contributed to the notable placebo response in this study. First, NERD is characterized by the absence of visible mucosal injury, which makes symptom assessment more subjective and prone to variability. This inherent variability, combined with psychological factors such as patient expectations, may have contributed to the relatively high placebo response. Furthermore, as NERD is driven by mechanisms beyond just acid reflux, including non-acid reflux and digestive enzymes, symptom relief can often be perceived in the absence of objective biomarkers, leading to higher placebo response rates. Second, the structured design of the clinical trial may have amplified the placebo response. Regular patient monitoring, the use of placebos closely resembling the active drug, and detailed symptom tracking likely heightened participants' expectations of improvement. These methodological elements, inherent to randomized controlled trials, play a significant role in enhancing placebo responses. Third, the 4-week treatment period may have magnified transient placebo effects, as short-term symptom fluctuations are more likely to be perceived as improvement. This brief observation window may have also limited the ability to observe long-term divergence in efficacy rates between the groups. Finally, the placebo used in this study contained a relatively high amount of cyclodextrin, which may have contributed to the observed placebo response. Cyclodextrins are known to form inclusion complexes with hydrophobic molecules, potentially reducing esophageal irritation caused by refluxate. Additionally, their mucoadhesive properties may enhance the mucosal barrier, providing a mild protective effect (49–52). These unintended effects, combined with the psychological impact of participating in a structured clinical trial, may have amplified the placebo response.

Despite the significant placebo response, JQ granules demonstrated superior efficacy compared to placebo (79.49% vs. 58.97%, *P* < 0.05), particularly in improving reflux symptoms, which were significantly better at both the 4-week treatment and 4-week follow-up periods. These results suggest that JQ granules have therapeutic potential in NERD, offering clinical benefits, especially in alleviating reflux symptoms. However, the heartburn response in this study was less distinct, and further studies with larger sample sizes and longer follow-up periods are warranted to further investigate the effects of JQ granules on heartburn relief and explore their long-term efficacy.

The challenges of treating GERD-related reflux symptoms with current therapies, particularly PPIs, highlight the potential advantages of JQ granules. Acid-suppressing therapy, while effective in reducing acidic reflux events, does not significantly decrease the occurrence of overall reflux events. Furthermore, it may transform acidic reflux into weak acid or alkaline reflux, which is often associated with a shift from heartburn to persistent reflux symptoms (53). Studies have shown that only 26%−44% of GERD patients with reflux as the main symptom respond to PPI treatment, with a response rate only 17% higher than placebo (47, 54).

Alternative therapies, such as baclofen, have been studied for PPI-refractory reflux. Baclofen, a gamma-aminobutyric acid B receptor agonist, reduces reflux events by inhibiting transient lower esophageal sphincter (LES) relaxations and increasing LES resting pressure. However, while it decreases the number of reflux events, it does not effectively improve patient symptoms and is associated with significant side effects, such as dizziness, drowsiness, and constipation, limiting its clinical use (55). Additionally, baclofen has not been approved by the U.S. Food and Drug Administration for the treatment of GERD, and long-term efficacy studies are lacking (56, 57). Consequently, refractory reflux symptoms that do not respond well to acid suppression therapy remain a significant challenge in GERD management.

The significant alleviation of reflux symptoms observed with JQ granules suggests that this treatment may offer distinct advantages, particularly for NERD patients with reflux-dominated symptoms. Unlike conventional therapies, JQ granules appear to address limitations associated with existing treatment options and may be especially beneficial for patients with non-acid reflux. Nonetheless, further research, particularly using multichannel intraluminal impedance-pH monitoring, is warranted to comprehensively evaluate the efficacy and mechanism of JQ granules in this context.

### Benefits of JQ granules for atypical symptoms in NERD

This study evaluated the effects of JQ granules on extraesophageal symptoms of NERD, such as cough and asthma. The placebo group showed no significant changes in cough frequency and severity scores from baseline (*P* > 0.05), whereas the JQ granules group demonstrated a significant reduction in these scores (*P* < 0.01). Similarly, asthma frequency scores increased in the placebo group compared to baseline (*P* > 0.05) but decreased in the JQ granules group, though this reduction did not reach statistical significance (*P* > 0.05). Despite the lack of statistically significant differences between the groups, the within-group analysis revealed a greater degree of improvement in both symptoms for the JQ granules group.

The observed improvement in respiratory symptoms in the JQ granules group corresponds with the reduction in reflux symptoms. Extraesophageal symptoms, particularly respiratory manifestations, are known to respond less effectively to acid-suppressive therapy compared to typical symptoms like reflux and heartburn. This may be due to the sensitivity of the upper airway structures, such as the throat, larynx, and pharynx, to non-acidic factors including weak acid reflux, weak alkaline reflux, gas reflux, pepsin, and bile salts. Research indicates that only 10% of extraesophageal symptoms are related to acid reflux, whereas up to 40% are associated with non-acid reflux events. Acid-suppressive agents, such as PPIs, reduce heartburn by lowering refluxate acidity but do not prevent non-acid reflux events or protect against airway aspiration (58–61).

The increased frequency of non-acid reflux events associated with acid-suppressive therapy may exacerbate respiratory symptoms through microaspiration and esophago-tracheo-bronchial reflexes (62). This study suggests that JQ granules may provide potential benefits for respiratory symptoms linked to NERD, potentially addressing the limitations of acid-suppressive therapy. However, their efficacy in treating PPI-refractory respiratory symptoms requires further validation in large-scale clinical studies using PPI as a positive control.

### Effect of JQ granules on TCM syndromes

This study demonstrated a significant improvement in TCM syndrome scores in the JQ granules group, with a 69.23% improvement rate compared to 46.15% in the placebo group after 4 weeks of treatment (*P* < 0.05). However, during the 4-week follow-up period, the efficacy rate in the JQ granules group declined to 53.85%, while the placebo group showed a slight increase to 56.41%. This unexpected trend, with no statistically significant difference between groups (*P* = 0.8978), may be explained by several factors.

First, the increase in the placebo group's efficacy rate during follow-up could be attributed to delayed placebo effects or natural symptom variability, both of which are common in NERD. Some placebo participants may have experienced transient symptom improvement due to natural fluctuations or psychological factors associated with their involvement in the trial. Second, the small sample size may have amplified these changes, as minor numerical shifts in patient responses can disproportionately affect percentage calculations. Lastly, the placebo composition, containing cyclodextrin, may have contributed to sustained symptom relief in some participants due to its potential mucosal protective effects. These findings underscore the importance of exploring longer-term treatment strategies, including the potential role of maintenance therapy with JQ granules.

Although the reduction in TCM syndrome scores after 4 weeks of treatment in the JQ granules group approached statistical significance compared to the placebo group (*P* = 0.0781), the results highlight the therapeutic potential of strengthening the spleen, clearing heat, and resolving dampness in managing the pathophysiological features of NERD. These findings align with the effectiveness of TCM strategies in alleviating symptoms associated with spleen deficiency and damp-heat syndrome.

Microscopic mucosal changes, despite the absence of visible mucosal erosions in NERD patients, are critical in the disease's pathophysiology. High-definition magnification endoscopy can detect subtle mucosal alterations, such as edema, indistinct vascular patterns, and patchy unevenness. Histological findings often reveal microscopic esophagitis, including basal cell hyperplasia, elongated papillae, intraepithelial inflammatory cell infiltration, and dilated intercellular spaces (DIS), which are hallmarks of diminished mucosal integrity (63).

The compromised mucosal barrier in NERD facilitates the penetration of noxious substances like pepsin and bile, triggering mast cell degranulation, histamine release, and peripheral sensitization (64, 65). DIS is prevalent in 68.2%−83% of NERD patients and plays a pivotal role in esophageal sensitivity to refluxants, including non-acidic or weakly acidic reflux. This sensitivity underscores the limited efficacy of PPI treatment in NERD patients with DIS and highlights the need for alternative approaches (66–73).

Consensus suggests that spleen damp-heat syndrome, manifesting in various aspects including the immune system, gastrointestinal motility, and microecology, is closely associated with inflammation (74). A large cross-sectional study (*n* = 1,000) indicates that the prevalence of microscopic mucosal changes in endoscopy among spleen damp-heat type NERD patients is significantly higher than in other syndrome types, implying a close relationship between spleen damp-heat syndrome and mucosal microscopic changes in NERD (75).

TCM's spleen damp-heat syndrome provides a theoretical basis for JQ granules' mechanism of action. Previous mechanistic studies have confirmed that JQ granules can enhance the proliferation and activation of mast cells in the esophageal mucosa of GERD models, reduce nerve ending sensitization, and improve mucosal barrier function (76). Although pathological histology was not included in this study, the symptom improvement observed with JQ granules suggests a potential role in addressing microscopic mucosal changes in spleen damp-heat type NERD patients.

Future research will focus on histopathological assessments of esophageal mucosa to elucidate the impact of JQ granules on microscopic lesions and their correlation with symptom improvement. This approach will help further validate the therapeutic benefits of JQ granules in NERD management and deepen the understanding of their mechanism of action.

### Effect of JQ granules on quality of life and patient-reported outcomes

The GERD-HRQL scale scores revealed no statistically significant differences between the JQ granules and placebo groups after 4 weeks of treatment or at the 4-week follow-up (*P* > 0.05). However, within-group analyses demonstrated a significant reduction in scores from baseline over time in both groups (JQ granules: *P* < 0.001; placebo: *P* < 0.01). Although the JQ granules group exhibited a more pronounced improvement compared to the placebo group, this difference did not reach statistical significance, potentially due to the limited sample size and the short treatment and follow-up durations.

The Chronic Gastrointestinal Disease PRO Scale, widely recognized for its reliability and validity in evaluating conditions such as NERD, revealed no statistically significant differences between the groups in scores for dyspepsia, bowel irregularity, psychological mood, or the total PRO Scale score at 4 weeks post-treatment and at the follow-up (*P* > 0.05). Despite this, there was a non-significant trend favoring the JQ granules group in the reflux and bowel irregularity dimensions, as well as in the total PRO Scale score.

For the reflux dimension, within-group analyses showed a significant reduction in scores from baseline in the JQ granules group at both 4 weeks post-treatment and the follow-up (*P* < 0.01). In contrast, the placebo group showed no significant change in reflux scores at the follow-up (*P* > 0.05). Between-group comparisons revealed a nearly statistically significant difference in reflux score improvements from baseline to 4 weeks post-treatment favoring the JQ granules group (*P* = 0.06735). This suggests a potential advantage of JQ granules in alleviating reflux symptoms, consistent with the main efficacy indicators.

In the social functioning dimension of the PRO Scale, the placebo group exhibited no significant improvement from baseline after 4 weeks of treatment (*P* > 0.05), whereas the JQ granules group demonstrated a significant improvement (*P* < 0.01). Between-group comparisons further revealed that the JQ granules group had significantly lower scores than the placebo group at both 4 weeks post-treatment and at the follow-up (*P* < 0.05), indicating that JQ granules may effectively enhance social functioning in NERD patients.

These findings suggest that JQ granules show promise in improving certain dimensions of quality of life, particularly social functioning and reflux-related symptoms.

### Effect of JQ granules on psychological conditions in NERD patients

This study examined the impact of JQ granules on psychological symptoms, such as anxiety and depression, in NERD patients. Both the JQ granules and placebo groups showed significant reductions in anxiety scores after 4 weeks of treatment and at the 4-week follow-up compared to baseline (*P* < 0.05 for both). However, the differences in anxiety scores between the two groups and the within-group changes in the JQ granules group did not reach statistical significance (*P* > 0.05).

For depression, within-group comparisons revealed no significant change in the placebo group's scores after treatment (*P* > 0.05), while the JQ granules group experienced a significant reduction in depression scores (*P* < 0.0001). Between-group comparisons showed that the JQ granules group's depression scores were lower than those of the placebo group, with the difference approaching statistical significance (*P* = 0.0607). These results suggest that JQ granules may have a beneficial effect on depressive symptoms in NERD patients.

Psychological symptoms, including anxiety and depression, are prevalent among GERD patients, with even higher rates in NERD patients compared to those with RE. In Chinese populations, the prevalence of anxiety and depression in NERD patients is reported at 51% and 45%, respectively (77). These symptoms exacerbate peripheral and central sensitivity, diminishing the effectiveness of acid suppression therapies and creating a cycle that worsens symptoms and quality of life, especially in NERD patients. Factors such as heightened sensitivity through the brain-gut axis and lowered sensory thresholds play crucial roles in NERD pathophysiology, with depression identified as a significant risk factor for worsening gastroesophageal reflux symptoms (78–82).

Emerging evidence links variations in gut microbiota composition to the interplay between GERD and psychological comorbidities. Firmicutes and Bacteroidetes are characteristic gut microbiota in NERD patients with psychological symptoms (83). JQ granules combined with low-dose PPI therapy (omeprazole 10 mg) have shown greater efficacy in improving psychological states compared to standard-dose PPI therapy (omeprazole 20 mg) (15). This combination therapy also significantly modulates the relative abundance of Firmicutes and Bacteroidetes, providing further evidence of the potential of JQ granules to alleviate psychological symptoms—particularly depressive symptoms—through the regulation of gut microbiota (15).

### Safety and overall efficacy of JQ granules in NERD treatment

This study confirmed the safety and efficacy of JQ granules in treating NERD. No significant effects on vital signs, the circulatory system, or liver and kidney function were observed, and no severe adverse events or complications occurred. Additionally, the incidence of adverse events was comparable between the JQ granules and placebo groups during treatment and follow-up (*P* > 0.05), highlighting the favorable safety profile of this herbal therapy.

In terms of efficacy, JQ granules demonstrated significant advantages over placebo, particularly in improving primary symptoms such as reflux. The treatment effectively addressed the pathophysiological features of NERD, showing greater efficacy in managing spleen deficiency and damp-heat syndrome compared to placebo. Furthermore, JQ granules exhibited trends of improvement in secondary symptoms, including TCM syndrome scores, GERD-HRQL scale scores, and the reflux and bowel irregularity dimensions of the PRO Scale, as well as anxiety and depression. The potential mechanisms underlying these effects involve the restoration of mucosal integrity and the modulation of gut microbiota. JQ granules enhance the esophageal mucosal barrier by promoting mast cell activation, reducing nerve sensitization, and mitigating esophageal permeability caused by DIS (76). These processes likely reduce sensitivity to refluxants, contributing to the relief of both acidic and non-acidic reflux symptoms. Additionally, by regulating the relative abundance of Firmicutes and Bacteroidetes, JQ granules may help restore gut microbiota balance (15). This effect is particularly relevant in NERD patients who present with psychological symptoms, as the modulation of gut microbiota is associated with improvements in both gastrointestinal and psychological outcomes.

### Future research directions

This study highlights the potential of JQ granules as a treatment for NERD, demonstrating significant efficacy in alleviating reflux symptoms. However, further research is needed to explore several key areas.

First, evaluating the efficacy of JQ granules in different subtypes of NERD (e.g., abnormal acid exposure, reflux hypersensitivity, and functional heartburn) will help tailor treatment strategies to specific patient populations. Investigating the impact of disease duration and using larger sample sizes will also enhance the generalizability of the findings, providing a clearer understanding of the long-term benefits of JQ granules. Additionally, combination therapies, particularly with low-dose PPIs, have shown promise in preliminary studies (15), and future trials should explore synergistic effects with other pharmacological agents, such as potassium-competitive acid blockers, antidepressants, and alginate, especially in patients with rGERD or psychological comorbidities.

While the findings from this study were promising, several factors may have influenced the observed efficacy and warrant further exploration. The small sample size, based on prior research estimates, may have limited the statistical power to detect significant differences. Increasing sample size in future trials would improve statistical robustness, particularly in heterogenous disorders like NERD, where symptom variability is more pronounced.

The impact of the COVID-19 pandemic on patient enrollment and follow-up data is another consideration. Disruptions caused by the pandemic may have introduced additional variability in the data, which could have affected the precision of the results. Future studies with extended follow-up periods and more consistent patient data collection are needed to assess long-term outcomes more accurately.

The high placebo response observed in this study, particularly for reflux symptoms, is common in NERD trials due to the functional nature of the disorder. Psychological factors, patient expectations, and natural symptom fluctuations play a substantial role in symptom relief. The placebo used in this study contained cyclodextrin, which may have further amplified the placebo response due to its mucoadhesive properties and potential to reduce esophageal irritation, suggesting that future studies should consider placebo formulations more carefully.

Finally, future research should include longer treatment and follow-up periods to capture the full therapeutic potential of JQ granules. Extended treatment durations would allow for sustained therapeutic effects and provide a better understanding of the long-term benefits. Further exploration into esophageal histopathology is essential to assess the impact of JQ granules on microscopic mucosal changes and their correlation with symptomatic improvement. Investigating molecular pathways related to mucosal barrier function and gut microbiota regulation will provide deeper insights into their mechanisms of action. Specific targets, including mucosal-specific activation protein kinase 2, substance P, and calcitonin gene-related peptide, may clarify how JQ granules alleviate NERD symptoms and improve patient outcomes.

## Conclusion

JQ granules have proven to be significantly more effective than a placebo in enhancing the overall efficacy rate of primary symptoms in the treatment of NERD. They exhibit superior long-term efficacy in managing reflux symptoms and effectively address the spleen deficiency and damp-heat syndrome typical of NERD. Furthermore, these granules have been shown to enhance social functioning more effectively than the placebo and exhibit a trend toward greater efficacy in improving the total frequency of atypical symptoms in NERD patients. JQ granules also demonstrate a positive trend in improving GERD-HRQL scores and PRO scale dimensions for reflux and bowel irregularity. They contribute to a reduction in anxiety and depression levels, with these benefits observed at the end of the treatment period and sustained during the follow-up phase. The safety profile of the granules in the treatment of NERD is commendable, indicating a reliable therapeutic option for patients.

## Data Availability

The raw data supporting the conclusions of this article will be made available by the authors, without undue reservation.

## Supplementary material

Supplementary Figure 1 Base Peak Ion (BPI) Chromatograms of the JQ Granule Test Solution. **(A)** Negative ion mode full-scan mass spectrometry. **(B)** Positive ion mode full-scan mass spectrometry. The structures of 164 compounds were preliminarily identified based on precursor ions, molecular formulas, MS/MS fragment ions, and retention times. Compound identification was further supported by reference literature and relevant databases (e.g., ChemSpider, PubChem).

Supplementary Figure 2 Changes in GERD-HRQL scores over the treatment and follow-up periods for both JQ granules and placebo groups. **(A)** GERD-HRQL scores. Both groups showed significant reductions from baseline levels after the 4-week treatment and at the follow-up (^**^*P* < 0.01, ^***^*P* < 0.001, and ^****^*P* < 0.0001). **(B)** Comparison of GERD-HRQL score changes post-treatment and at follow-up. No significant differences were observed between the groups (ns, not significant; *P* > 0.05). Both groups exhibited a consistent downward trend in scores, with a non-significant trend suggesting a slight advantage for the JQ granules group at the 4-week follow-up. Statistical significance is denoted by ^*^*P* < 0.05, ^**^*P* < 0.01, ^***^*P* < 0.001, and ^****^*P* < 0.0001.

Supplementary Figure 3 Changes in scores for dyspepsia, bowel irregularity, psychological mood, reflux dimension, systemic symptom dimension, social functioning dimension, and total scores of the Chronic Gastrointestinal Disease PRO Scale over the treatment and follow-up periods for both JQ granules (red bars) and placebo groups (blue bars). **(A)** Dyspepsia scores. Both groups showed significant reductions from baseline after the 4-week treatment and at the 4-week follow-up (^****^*P* < 0.0001 for both groups). **(B)** Bowel irregularity scores. Significant reductions were observed in both groups from baseline after the 4-week treatment and at the 4-week follow-up (^***^*P* < 0.001; ^****^*P* < 0.0001). **(C)** Psychological mood scores. Both groups exhibited significant improvements from baseline at both the post-treatment and follow-up assessments (^***^*P* < 0.001; ^****^*P* < 0.0001). **(D)** Reflux dimension scores. The JQ granules group showed a significant decrease from baseline after the 4-week treatment (^***^*P* < 0.001) and maintained this decrease at the follow-up (^**^*P* < 0.01). The placebo group also showed a significant decrease after the 4-week treatment (^*^*P* < 0.05) but did not maintain this at the follow-up (ns, not significant; *P* > 0.05). **(E)** Systemic symptom dimension scores. The placebo group exhibited a significant reduction after the 4-week treatment (^*^*P* < 0.05). There was no significant difference for the JQ granules group at the end of the 4-week treatment (ns, not significant; *P* > 0.05). Both groups showed significant reductions at the 4-week follow-up (^*^*P* < 0.05 for JQ granules, ^***^*P* < 0.001 for placebo). **(F)** Social functioning dimension scores. The JQ granules group showed significant reductions after the 4-week treatment (^**^*P* < 0.01) and at the follow-up (^**^*P* < 0.01). The placebo group showed significant reductions at the follow-up (^**^*P* < 0.01), but not at the post-treatment stage (ns, not significant; *P* > 0.05). **(G)** Total PRO Scale scores. Both groups exhibited significant reductions from baseline after the 4-week treatment and at the follow-up (^****^*P* < 0.0001 for both groups). Statistical significance is denoted by ^*^*P* < 0.05, ^**^*P* < 0.01, ^***^*P* < 0.001, and ^****^*P* < 0.0001.

Supplementary Figure 4 Comparison of scores for dyspepsia, bowel irregularity, psychological mood, reflux dimension, systemic symptom dimension, social functioning dimension, and total scores of the Chronic Gastrointestinal Disease PRO Scale between JQ granules (red line) and placebo groups (blue line) over the treatment and follow-up periods. **(A)** Dyspepsia scores. No significant differences were observed between the groups at the post-treatment or follow-up assessments (ns, not significant; *P* > 0.05). Both groups showed a consistent downward trend across all measured time points. **(B)** Bowel irregularity scores. No significant differences were observed between the groups at the post-treatment or follow-up assessments (ns, not significant; *P*> 0.05). A non-significant trend suggests that the JQ granules group may have had marginally better outcomes. **(C)** Psychological mood scores. No significant differences were observed between the groups at the post-treatment or follow-up assessments (ns, not significant; *P*> 0.05). Both groups exhibited a consistent downward trend in scores. **(D)** Reflux dimension scores. No significant differences were found between the JQ granules and placebo groups at either the 4-week post-treatment or the 4-week follow-up (ns, not significant; *P*> 0.05). A non-significant trend suggests the JQ granules group may have performed slightly better. **(E)** Systemic symptom dimension scores. No significant differences were observed between the two groups at either the 4-week post-treatment or the 4-week follow-up assessments (ns, not significant; *P*> 0.05). Both groups showed a consistent downward trend in scores. **(F)** Social functioning dimension scores. The JQ granules group had significantly lower scores than the placebo group at both the 4-week post-treatment and the 4-week follow-up (^*^*P* < 0.05). Both groups exhibited a downward trend in scores. **(G)** Total PRO Scale scores. No statistically significant differences were observed between the JQ granules and placebo groups at the 4-week post-treatment or the follow-up (ns, not significant; *P*> 0.05). A non-significant trend suggests that the JQ granules group may have shown a better improvement. Each panel demonstrates the trends over time for both treatment groups, highlighting improvements within groups and indicating potential marginal advantages of JQ granules over placebo in certain dimensions. Statistical significance is denoted by ^*^*P* < 0.05, ^**^*P* < 0.01, ^***^*P* < 0.001, and ^****^*P* < 0.0001.
