# Supplementary figures and images for: Efficacy and safety of Jianpi Qinghua granules for non-erosive reflux disease with spleen deficiency and damp-heat syndrome: a multicenter, randomized, double-blind, placebo-controlled clinical trial
Source: Front Nutr. 2025 Jan 7;11:1509931. doi: 10.3389/fnut.2024.1509931 (PMC11747786; doi:10.3389/fnut.2024.1509931)

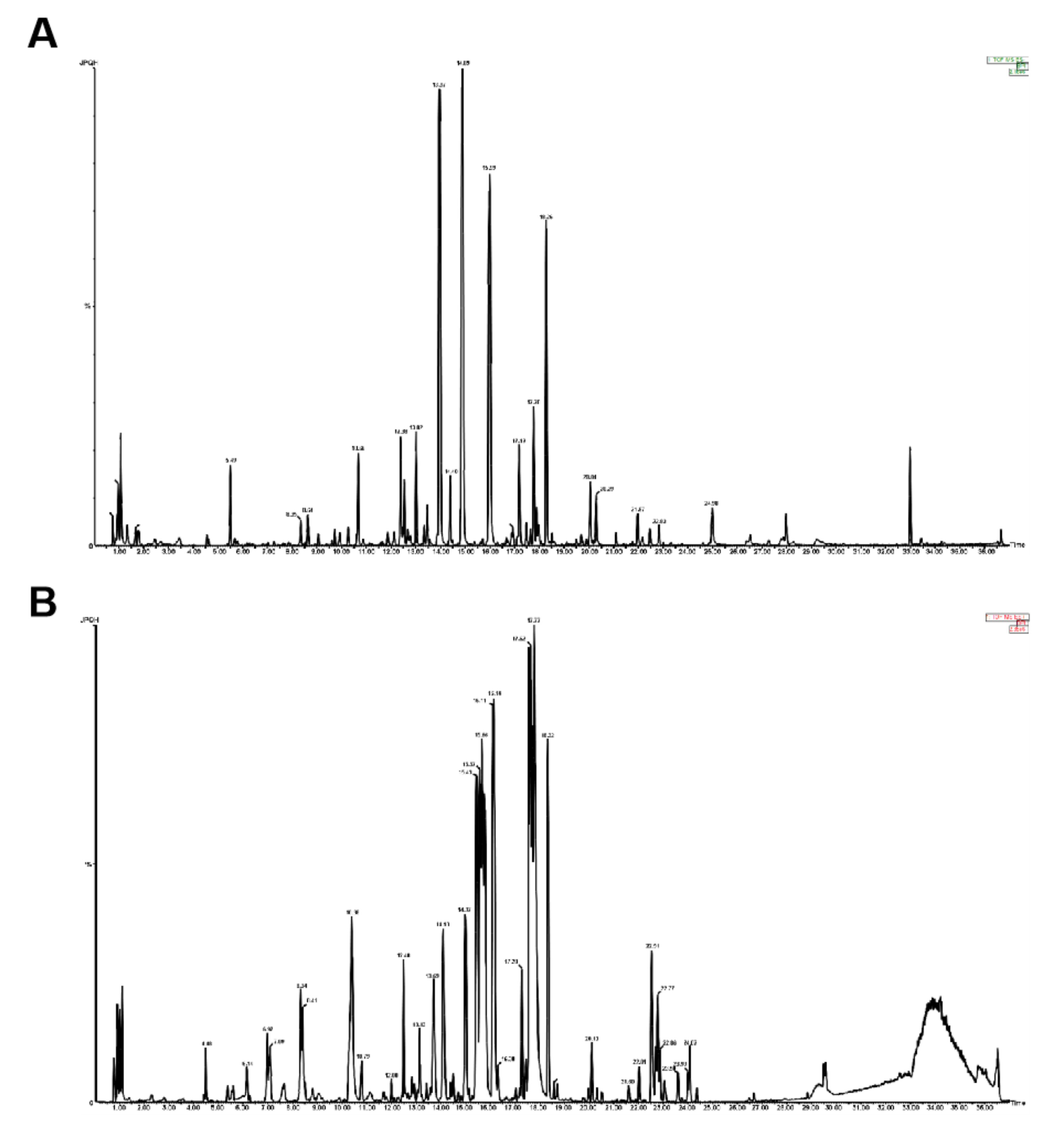

Supplement: Supplementary Figure 1 — Base Peak Ion (BPI) Chromatograms of the JQ Granule Test Solution. (A) Negative ion mode full-scan mass spectrometry. (B) Positive ion mode full-scan mass spectrometry. The structures of 164 compounds were preliminarily identified based on precursor ions, molecular formulas, MS/MS fragment ions, and retention times. Compound identification was further supported by reference literature and relevant databases (e.g., ChemSpider, PubChem). [file Image_1.jpeg]

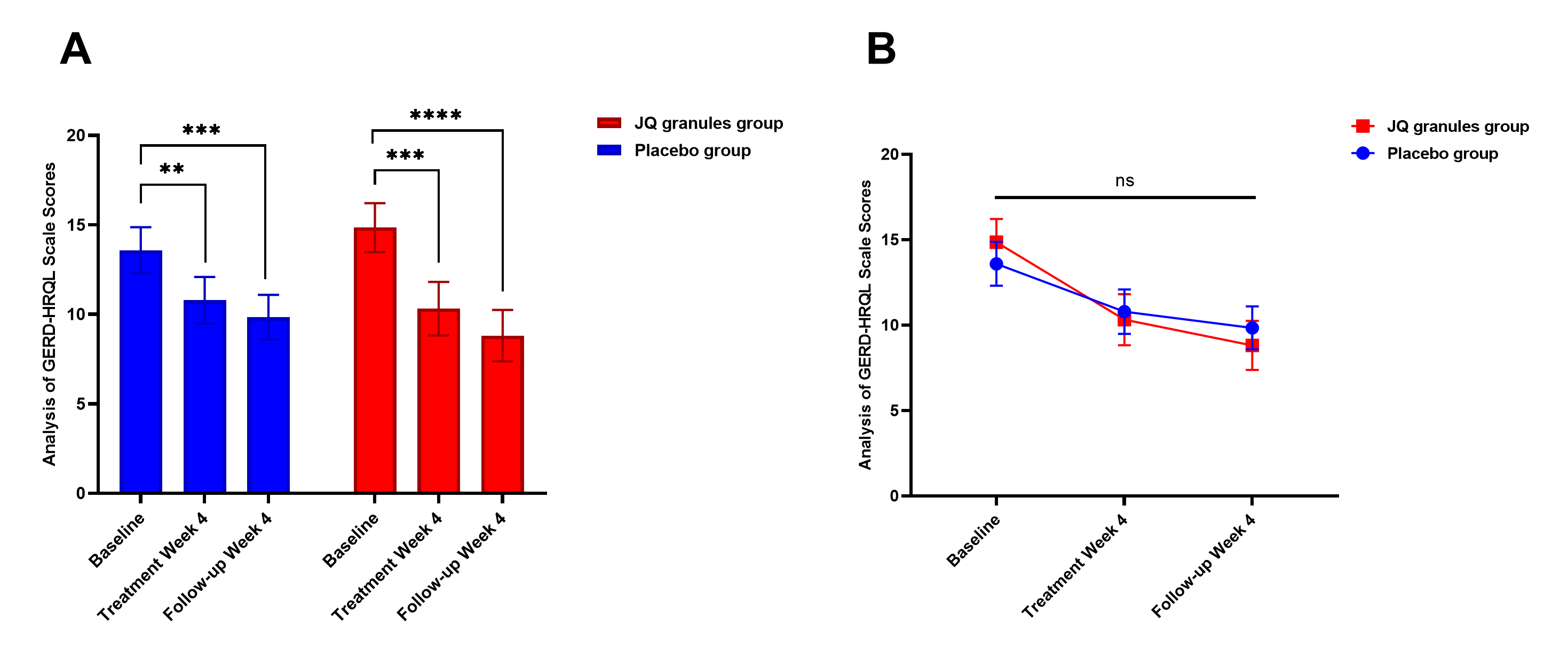

Supplement: Supplementary Figure 2 — Changes in GERD-HRQL scores over the treatment and follow-up periods for both JQ granules and placebo groups. (A) GERD-HRQL scores. Both groups showed significant reductions from baseline levels after the 4-week treatment and at the follow-up (**P < 0.01, ***P < 0.001, and ****P < 0.0001). (B) Comparison of GERD-HRQL score changes post-treatment and at follow-up. No significant differences were observed between the groups (ns, not significant; P > 0.05). Both groups exhibited a consistent downward trend in scores, with a non-significant trend suggesting a slight advantage for the JQ granules group at the 4-week follow-up. Statistical significance is denoted by *P < 0.05, **P < 0.01, ***P < 0.001, and ****P < 0.0001. [file Image_2.tif]

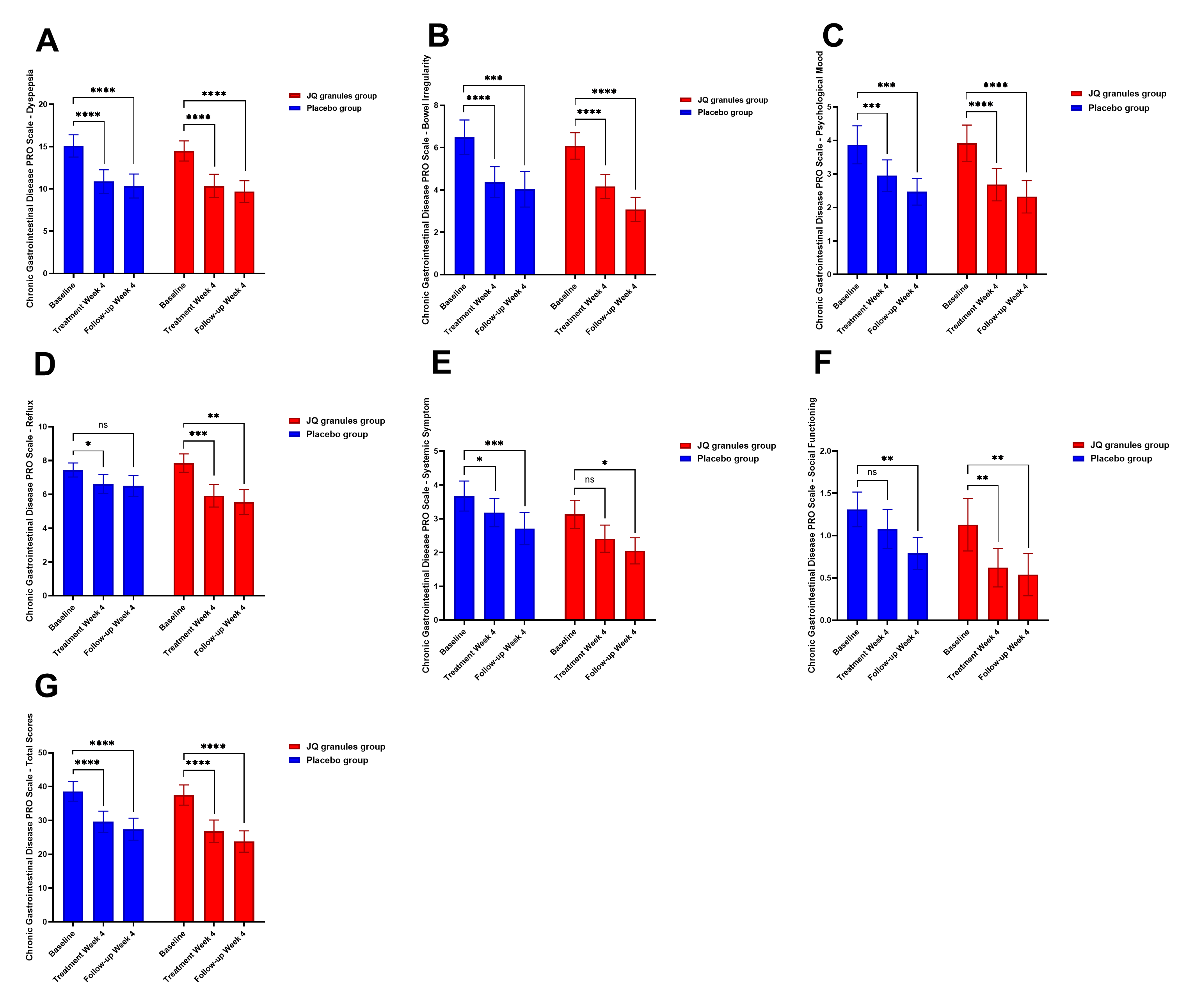

Supplement: Supplementary Figure 3 — Changes in scores for dyspepsia, bowel irregularity, psychological mood, reflux dimension, systemic symptom dimension, social functioning dimension, and total scores of the Chronic Gastrointestinal Disease PRO Scale over the treatment and follow-up periods for both JQ granules (red bars) and placebo groups (blue bars). (A) Dyspepsia scores. Both groups showed significant reductions from baseline after the 4-week treatment and at the 4-week follow-up (****P < 0.0001 for both groups). (B) Bowel irregularity scores. Significant reductions were observed in both groups from baseline after the 4-week treatment and at the 4-week follow-up (***P < 0.001; ****P < 0.0001). (C) Psychological mood scores. Both groups exhibited significant improvements from baseline at both the post-treatment and follow-up assessments (***P < 0.001; ****P < 0.0001). (D) Reflux dimension scores. The JQ granules group showed a significant decrease from baseline after the 4-week treatment (***P < 0.001) and maintained this decrease at the follow-up (**P < 0.01). The placebo group also showed a significant decrease after the 4-week treatment (*P < 0.05) but did not maintain this at the follow-up (ns, not significant; P > 0.05). (E) Systemic symptom dimension scores. The placebo group exhibited a significant reduction after the 4-week treatment (*P < 0.05). There was no significant difference for the JQ granules group at the end of the 4-week treatment (ns, not significant; P > 0.05). Both groups showed significant reductions at the 4-week follow-up (*P < 0.05 for JQ granules, ***P < 0.001 for placebo). (F) Social functioning dimension scores. The JQ granules group showed significant reductions after the 4-week treatment (**P < 0.01) and at the follow-up (**P < 0.01). The placebo group showed significant reductions at the follow-up (**P < 0.01), but not at the post-treatment stage (ns, not significant; P > 0.05). (G) Total PRO Scale scores. Both groups exhibited significant reductions from baseline a [file Image_3.tif]

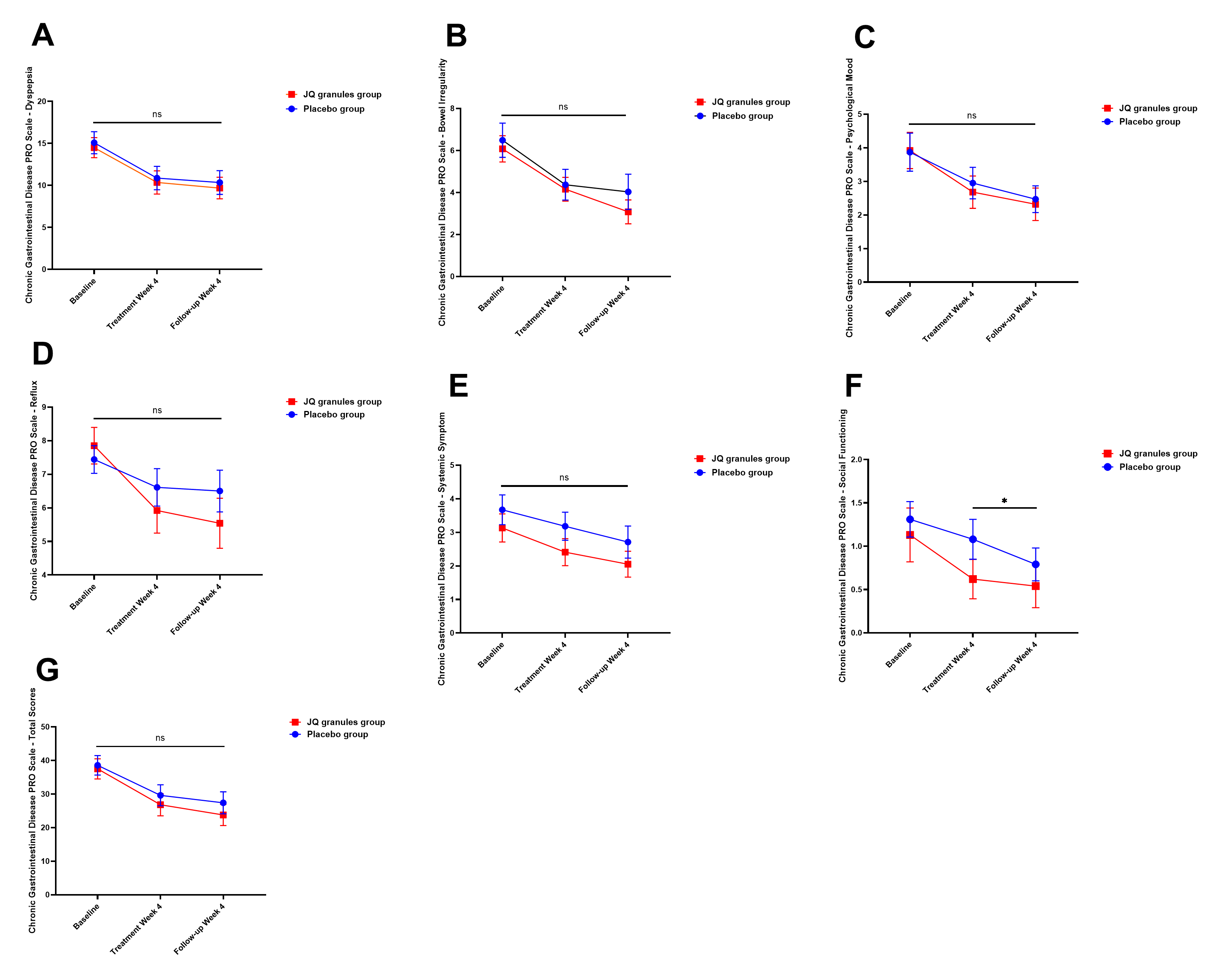

Supplement: Supplementary Figure 4 — Comparison of scores for dyspepsia, bowel irregularity, psychological mood, reflux dimension, systemic symptom dimension, social functioning dimension, and total scores of the Chronic Gastrointestinal Disease PRO Scale between JQ granules (red line) and placebo groups (blue line) over the treatment and follow-up periods. (A) Dyspepsia scores. No significant differences were observed between the groups at the post-treatment or follow-up assessments (ns, not significant; P > 0.05). Both groups showed a consistent downward trend across all measured time points. (B) Bowel irregularity scores. No significant differences were observed between the groups at the post-treatment or follow-up assessments (ns, not significant; P> 0.05). A non-significant trend suggests that the JQ granules group may have had marginally better outcomes. (C) Psychological mood scores. No significant differences were observed between the groups at the post-treatment or follow-up assessments (ns, not significant; P> 0.05). Both groups exhibited a consistent downward trend in scores. (D) Reflux dimension scores. No significant differences were found between the JQ granules and placebo groups at either the 4-week post-treatment or the 4-week follow-up (ns, not significant; P> 0.05). A non-significant trend suggests the JQ granules group may have performed slightly better. (E) Systemic symptom dimension scores. No significant differences were observed between the two groups at either the 4-week post-treatment or the 4-week follow-up assessments (ns, not significant; P> 0.05). Both groups showed a consistent downward trend in scores. (F) Social functioning dimension scores. The JQ granules group had significantly lower scores than the placebo group at both the 4-week post-treatment and the 4-week follow-up (*P < 0.05). Both groups exhibited a downward trend in scores. (G) Total PRO Scale scores. No statistically significant differences were observed between the JQ granules and placebo groups at the 4-w [file Image_4.tif]
