# Supplementary material for: Efficacy and safety of Jianpi Qinghua granules for non-erosive reflux disease with spleen deficiency and damp-heat syndrome: a multicenter, randomized, double-blind, placebo-controlled clinical trial
Source: Front Nutr. 2025 Jan 7;11:1509931. doi: 10.3389/fnut.2024.1509931 (PMC11747786; doi:10.3389/fnut.2024.1509931)
Supplement: Supplementary file 5 [file Table_1.docx]

**Supplementary Table 1**

TCM diagnostic criteria for spleen deficiency with damp-heat syndrome in NERD.

| TCM diagnostic criteria for spleen deficiency with damp-heat syndrome in NERD | |
| --- | --- |
| Major Symptoms | 1. Acid reflux following meals |
|  | 1. Abdominal bloating |
| Minor Symptoms | 1. Burning sensation in the epigastric area |
|  | 1. Chest tightness and discomfort |
|  | 1. Loss of appetite |
|  | 1. General fatigue |
|  | 1. Loose and sluggish stool |
| Tongue and Pulse Characteristics | 1. Pale or reddish tongue with a thin, yellow, greasy coating |
|  | 1. Pulse that is thin, slippery, and rapid |

**Supplementary Table 2**

Mass Spectrometric Analysis of Compounds Identified in JQ Granules by UPLC-ESI-QTOF-MS/MS.

| **No.** | **Time (min)** | **Theoretical Mass (Da)** | **Measured Mass (Da)** | **Error (ppm)** | **Identified Compound** | **Source** |
| --- | --- | --- | --- | --- | --- | --- |
| 1 | 0.81 | 296.1987 | 296.1989 | 0.4 | 6-Gingediol | *Zingiber officinale* Roscoe [Zingiberaceae; Zingiberis Rhizoma] |
| 2 | 0.81 | 470.194 | 470.1868 | -14.3 | Obaculactone | *Citrus aurantium* L. [Rutaceae; Aurantii Fructus] |
| 3 | 0.82 | 268.1548 | 268.1486 | -21.7 | Codonopyrrolidium B | *Codonopsis pilosula* (Franch.) Nannf. [Campanulaceae; Codonopsis Radix] |
| 4 | 0.85 | 1014.5399 | 1014.5438 | 3.8 | Codonolaside II | *Codonopsis pilosula* (Franch.) Nannf. [Campanulaceae; Codonopsis Radix] |
| 5 | 0.86 | 246.1255 | 246.1314 | 21.6 | Zederone | *Atractylodes lancea* (Thunb.) DC. [Asteraceae; Atractylodis Rhizoma] |
| 6 | 0.86 | 1206.5669 | 1206.5608 | -5.1 | Lancemaside G | *Codonopsis pilosula* (Franch.) Nannf. [Campanulaceae; Codonopsis Radix] |
| 7 | 0.86 | 1204.5876 | 1204.5659 | -17.4 | Codonolaside I | *Codonopsis pilosula* (Franch.) Nannf. [Campanulaceae; Codonopsis Radix] |
| 8 | 0.86 | 244.1099 | 244.1041 | -22.2 | Osthol | *Atractylodes lancea* (Thunb.) DC. [Asteraceae; Atractylodis Rhizoma] |
| 9 | 0.86 | 1088.5403 | 1088.5131 | -25 | Lancemaside D | *Codonopsis pilosula* (Franch.) Nannf. [Campanulaceae; Codonopsis Radix] |
| 10 | 0.9 | 288.0633 | 288.0781 | 44.3 | Carthamidin | *Scutellaria baicalensis* Georgi [Lamiaceae; Scutellariae Radix] |
| 11 | 0.93 | 126.0316 | 126.0336 | 11.4 | 5-Hydroxymethylfuraldehyde | *Codonopsis pilosula* (Franch.) Nannf. [Campanulaceae; Codonopsis Radix] |
| 12 | 0.95 | 302.079 | 302.093 | 40.3 | 5,7,4'-Trihydroxy-6-methoxyflavanone | *Scutellaria baicalensis* Georgi [Lamiaceae; Scutellariae Radix] |
| 13 | 0.95 | 376.0794 | 376.0827 | 7.8 | 5,7,2',5'-Tetrahydroxy-8,6-dimethoxyflavone | *Scutellaria baicalensis* Georgi [Lamiaceae; Scutellariae Radix] |
| 14 | 0.96 | 295.1055 | 295.111 | 18.4 | Prunasin | *Perilla frutescens* (L.) Britton [Lamiaceae; Perillae Folium] |
| 15 | 0.96 | 346.0688 | 346.0626 | -18 | Eupatolitin | *Citrus aurantium* L. [Rutaceae; Aurantii Fructus] |
| 16 | 1.07 | 843.1983 | 843.2294 | 36.7 | Malonylshisonin | *Perilla frutescens* (L.) Britton [Lamiaceae; Perillae Folium] |
| 17 | 1.07 | 338.1392 | 338.1345 | -12.3 | Columbamine | *Coptis chinensis* Franch. [Ranunculaceae; Coptidis Rhizoma] |
| 18 | 1.09 | 388.1369 | 388.1347 | -5.8 | Geniposide | *Atractylodes lancea* (Thunb.) DC. [Asteraceae; Atractylodis Rhizoma] |
| 19 | 1.14 | 592.1792 | 592.1764 | -4.4 | Linarin | *Scutellaria baicalensis* Georgi [Lamiaceae; Scutellariae Radix] |
| 20 | 1.32 | 316.1674 | 316.1736 | 18.2 | Atractyloyne | *Atractylodes lancea* (Thunb.) DC. [Asteraceae; Atractylodis Rhizoma] |
| 21 | 1.75 | 208.1099 | 208.1084 | -7.4 | Elemicin | *Perilla frutescens* (L.) Britton [Lamiaceae; Perillae Folium] |
| 22 | 2.62 | 224.214 | 224.2216 | 28.9 | Eudesmol | *Atractylodes lancea* (Thunb.) DC. [Asteraceae; Atractylodis Rhizoma] |
| 23 | 3.09 | 305.199 | 305.1936 | -16.1 | Capsaicin | *Zingiber officinale* Roscoe [Zingiberaceae; Zingiberis Rhizoma] |
| 24 | 4.4 | 128.1201 | 128.1185 | -9.8 | Matsutake alcohol | *Perilla frutescens* (L.) Britton [Lamiaceae; Perillae Folium] |
| 25 | 4.59 | 372.12 | 372.117 | -9.3 | Sinensetin | *Citrus aurantium* L. [Rutaceae; Aurantii Fructus] |
| 26 | 4.63 | 360.0845 | 360.0848 | 0.7 | Rosmarinic acid | *Scutellaria baicalensis* Georgi [Lamiaceae; Scutellariae Radix] |
| 27 | 4.74 | 342.1705 | 342.1782 | 22.4 | Magnoflorine | *Coptis chinensis* Franch. [Ranunculaceae; Coptidis Rhizoma] |
| 28 | 4.79 | 166.0993 | 166.0984 | -4.8 | Perilla ketone | *Perilla frutescens* (L.) Britton [Lamiaceae; Perillae Folium] |
| 29 | 5.23 | 156.1514 | 156.1495 | -10 | Menthol | *Perilla frutescens* (L.) Britton [Lamiaceae; Perillae Folium] |
| 30 | 5.59 | 264.1361 | 264.1397 | 11.4 | Plenolin | *Perilla frutescens* (L.) Britton [Lamiaceae; Perillae Folium] |
| 31 | 5.72 | 154.1357 | 154.1339 | -9.8 | (R)-Linalool | *Perilla frutescens* (L.) Britton [Lamiaceae; Perillae Folium] |
| 32 | 5.9 | 370.1416 | 370.146 | 11.7 | 1,2-Dihydrocurcumin | *Zingiber officinale* Roscoe [Zingiberaceae; Zingiberis Rhizoma] |
| 33 | 5.99 | 207.0895 | 207.089 | -2.6 | Corydaldine | *Coptis chinensis* Franch. [Ranunculaceae; Coptidis Rhizoma] |
| 34 | 6.48 | 402.1314 | 402.128 | -8.5 | Nobiletin | *Citrus aurantium* L. [Rutaceae; Aurantii Fructus] |
| 35 | 6.79 | 420.1784 | 420.1675 | -23.8 | Eupaformosanin | *Eupatorium fortunei* Turcz. [Asteraceae; Eupatorii Herba] |
| 36 | 6.85 | 450.246 | 450.2519 | 10.9 | Atractyloside B | *Atractylodes lancea* (Thunb.) DC. [Asteraceae; Atractylodis Rhizoma] |
| 37 | 7.07 | 198.068 | 198.0688 | 3.1 | Atractylodinol | *Atractylodes lancea* (Thunb.) DC. [Asteraceae; Atractylodis Rhizoma] |
| 38 | 7.15 | 448.1005 | 448.1055 | 11 | Galuteolin | *Scutellaria baicalensis* Georgi [Lamiaceae; Scutellariae Radix] |
| 39 | 7.25 | 286.0477 | 286.0568 | 31.5 | Scutellarein | *Scutellaria baicalensis* Georgi [Lamiaceae; Scutellariae Radix] |
| 40 | 7.28 | 448.2308 | 448.2358 | 10.1 | 10-epi-Atractyloside A | *Atractylodes lancea* (Thunb.) DC. [Asteraceae; Atractylodis Rhizoma] |
| 41 | 7.87 | 618.236 | 618.2352 | -2.3 | Oxofangchirine | *Coptis chinensis* Franch. [Ranunculaceae; Coptidis Rhizoma] |
| 42 | 8.1 | 248.1412 | 248.1453 | 13.7 | Atractylenolide III | *Atractylodes lancea* (Thunb.) DC. [Asteraceae; Atractylodis Rhizoma] |
| 43 | 8.17 | 322.1079 | 322.1155 | 23.5 | Groenlandicine | *Coptis chinensis* Franch. [Ranunculaceae; Coptidis Rhizoma] |
| 44 | 8.32 | 392.1471 | 392.142 | -13.1 | Eupatoroxin | *Eupatorium fortunei* Turcz. [Asteraceae; Eupatorii Herba] |
| 45 | 8.37 | 350.1967 | 350.2072 | 26.5 | Codonopyrrolidium A | *Codonopsis pilosula* (Franch.) Nannf. [Campanulaceae; Codonopsis Radix] |
| 46 | 8.39 | 293.1415 | 293.1435 | 6.6 | Stephanthrine | *Coptis chinensis* Franch. [Ranunculaceae; Coptidis Rhizoma] |
| 47 | 8.43 | 314.079 | 314.0829 | 12.4 | Panicolin | *Scutellaria baicalensis* Georgi [Lamiaceae; Scutellariae Radix] |
| 48 | 8.51 | 365.1474 | 365.1599 | 34 | Erucifoline N-oxide | *Eupatorium fortunei* Turcz. [Asteraceae; Eupatorii Herba] |
| 49 | 8.55 | 726.2735 | 726.3095 | 49.6 | Tangshenoside III | *Codonopsis pilosula* (Franch.) Nannf. [Campanulaceae; Codonopsis Radix] |
| 50 | 8.55 | 272.0684 | 272.0774 | 32.7 | Naringenin | *Citrus aurantium* L. [Rutaceae; Aurantii Fructus] |
| 51 | 8.55 | 362.1729 | 362.1655 | -18.4 | Eupatoriopicrin | *Eupatorium fortunei* Turcz. [Asteraceae; Eupatorii Herba] |
| 52 | 8.64 | 344.0896 | 344.1012 | 29.9 | Rivularin | *Scutellaria baicalensis* Georgi [Lamiaceae; Scutellariae Radix] |
| 53 | 8.64 | 351.1106 | 351.1174 | 19.2 | Oxyberberine | *Coptis chinensis* Franch. [Ranunculaceae; Coptidis Rhizoma] |
| 54 | 9.39 | 334.178 | 334.1847 | 18.9 | Arnicolide C | *Atractylodes lancea* (Thunb.) DC. [Asteraceae; Atractylodis Rhizoma] |
| 55 | 9.54 | 302.079 | 302.0871 | 26.6 | Hesperetin | *Citrus aurantium* L. [Rutaceae; Aurantii Fructus] |
| 56 | 9.63 | 374.1729 | 374.1725 | -1 | Hexahydrocurcumin | *Zingiber officinale* Roscoe [Zingiberaceae; Zingiberis Rhizoma] |
| 57 | 9.74 | 594.1584 | 594.1548 | -6.1 | Lonicerin | *Citrus aurantium* L. [Rutaceae; Aurantii Fructus] |
| 58 | 9.87 | 306.1467 | 306.1473 | 1.6 | Eupaformonin | *Eupatorium fortunei* Turcz. [Asteraceae; Eupatorii Herba] |
| 59 | 10.21 | 320.0922 | 320.1002 | 24.6 | Coptisine | *Coptis chinensis* Franch. [Ranunculaceae; Coptidis Rhizoma] |
| 60 | 10.27 | 325.1525 | 325.1491 | -10.5 | Monocrotaline | *Atractylodes lancea* (Thunb.) DC. [Asteraceae; Atractylodis Rhizoma] |
| 61 | 10.27 | 341.1838 | 341.1796 | -12.6 | 7-Acetylintermedine | *Eupatorium fortunei* Turcz. [Asteraceae; Eupatorii Herba] |
| 62 | 10.27 | 376.1522 | 376.1461 | -16.3 | Euparotin | *Eupatorium fortunei* Turcz. [Asteraceae; Eupatorii Herba] |
| 63 | 10.36 | 338.1392 | 338.1461 | 20.4 | Jatrorrhizine | *Coptis chinensis* Franch. [Ranunculaceae; Coptidis Rhizoma] |
| 64 | 10.37 | 320.1987 | 320.2007 | 5.6 | 8-Gingerdion | *Zingiber officinale* Roscoe [Zingiberaceae; Zingiberis Rhizoma] |
| 65 | 10.44 | 346.0688 | 346.0757 | 19.6 | Viscidulin III | *Scutellaria baicalensis* Georgi [Lamiaceae; Scutellariae Radix] |
| 66 | 10.49 | 336.1235 | 336.1306 | 20.8 | Epiberberine | *Coptis chinensis* Franch. [Ranunculaceae; Coptidis Rhizoma] |
| 67 | 10.63 | 432.1056 | 432.1102 | 10.5 | Cosmetin | *Perilla frutescens* (L.) Britton [Lamiaceae; Perillae Folium] |
| 68 | 10.67 | 367.1631 | 367.1741 | 30.1 | Retrorsine N-oxide | *Eupatorium fortunei* Turcz. [Asteraceae; Eupatorii Herba] |
| 69 | 10.7 | 270.0528 | 270.0623 | 34.8 | Baicalein | *Scutellaria baicalensis* Georgi [Lamiaceae; Scutellariae Radix] |
| 70 | 10.83 | 474.1162 | 474.1005 | -33.3 | Apigenin-7-O-glucuronide-6'-ethylester | *Scutellaria baicalensis* Georgi [Lamiaceae; Scutellariae Radix] |
| 71 | 10.84 | 239.1157 | 239.1148 | -3.9 | Codonopsinol C | *Codonopsis pilosula* (Franch.) Nannf. [Campanulaceae; Codonopsis Radix] |
| 72 | 10.88 | 229.0738 | 229.0726 | -5.2 | Fagarine | *Coptis chinensis* Franch. [Ranunculaceae; Coptidis Rhizoma] |
| 73 | 10.99 | 191.0582 | 191.0597 | 7.8 | Noroxyhydrastinine | *Coptis chinensis* Franch. [Ranunculaceae; Coptidis Rhizoma] |
| 74 | 11.11 | 678.2371 | 678.2247 | -18.3 | Tangshenoside I | *Codonopsis pilosula* (Franch.) Nannf. [Campanulaceae; Codonopsis Radix] |
| 75 | 11.18 | 230.1306 | 230.1266 | -14.9 | (+)-Atractylenolide | *Atractylodes lancea* (Thunb.) DC. [Asteraceae; Atractylodis Rhizoma] |
| 76 | 11.55 | 448.1005 | 448.1055 | 10.9 | Dihydrobaicalin | *Scutellaria baicalensis* Georgi [Lamiaceae; Scutellariae Radix] |
| 77 | 11.6 | 576.2781 | 576.2709 | -11.7 | Atractyloside I | *Atractylodes lancea* (Thunb.) DC. [Asteraceae; Atractylodis Rhizoma] |
| 78 | 11.71 | 334.1079 | 334.1165 | 25.7 | Worenine | *Coptis chinensis* Franch. [Ranunculaceae; Coptidis Rhizoma] |
| 79 | 11.87 | 622.117 | 622.11 | -11.2 | Apigenin 7-O-diglucuronide | *Scutellaria baicalensis* Georgi [Lamiaceae; Scutellariae Radix] |
| 80 | 12.19 | 345.1787 | 345.1708 | -23.2 | Europine N-oxide | *Eupatorium fortunei* Turcz. [Asteraceae; Eupatorii Herba] |
| 81 | 12.32 | 216.0786 | 216.0678 | -46.3 | Euparin | *Eupatorium fortunei* Turcz. [Asteraceae; Eupatorii Herba] |
| 82 | 12.33 | 352.1548 | 352.1628 | 22.5 | Palmatine | *Coptis chinensis* Franch. [Ranunculaceae; Coptidis Rhizoma] |
| 83 | 12.36 | 372.142 | 372.131 | -26.3 | Syringin | *Codonopsis pilosula* (Franch.) Nannf. [Campanulaceae; Codonopsis Radix] |
| 84 | 12.42 | 167.0946 | 167.0874 | -38.2 | Synephrine | *Citrus aurantium* L. [Rutaceae; Aurantii Fructus] |
| 85 | 12.43 | 336.1235 | 336.1313 | 23 | Berberine | *Coptis chinensis* Franch. [Ranunculaceae; Coptidis Rhizoma] |
| 86 | 12.47 | 608.2886 | 608.2962 | 12.4 | Oxyacanthine | *Coptis chinensis* Franch. [Ranunculaceae; Coptidis Rhizoma] |
| 87 | 12.67 | 462.0798 | 462.0862 | 13.9 | Scutellarin | *Scutellaria baicalensis* Georgi [Lamiaceae; Scutellariae Radix] |
| 88 | 12.69 | 254.0579 | 254.0678 | 38.9 | Chrysin | *Scutellaria baicalensis* Georgi [Lamiaceae; Scutellariae Radix] |
| 89 | 12.92 | 328.1522 | 328.1654 | 35.3 | Perilloside B | *Perilla frutescens* (L.) Britton [Lamiaceae; Perillae Folium] |
| 90 | 12.93 | 396.1784 | 396.1868 | 19 | Lobetyolin | *Codonopsis pilosula* (Franch.) Nannf. [Campanulaceae; Codonopsis Radix] |
| 91 | 13.18 | 346.0688 | 346.0548 | -38.5 | Ganhuangenin | *Scutellaria baicalensis* Georgi [Lamiaceae; Scutellariae Radix] |
| 92 | 13.26 | 464.1318 | 464.1386 | 14.6 | Hesperetin 7-O-β-D-glucoside | *Citrus aurantium* L. [Rutaceae; Aurantii Fructus] |
| 93 | 13.4 | 558.2312 | 558.2256 | -9.4 | Lobetyolinin | *Codonopsis pilosula* (Franch.) Nannf. [Campanulaceae; Codonopsis Radix] |
| 94 | 13.49 | 328.0946 | 328.1025 | 23.8 | Salvigenin | *Scutellaria baicalensis* Georgi [Lamiaceae; Scutellariae Radix] |
| 95 | 13.57 | 578.1635 | 578.1922 | 45.9 | Rhoifolin | *Citrus aurantium* L. [Rutaceae; Aurantii Fructus] |
| 96 | 13.89 | 859.1933 | 859.178 | -17.9 | Caffeylmalonylcyanin | *Perilla frutescens* (L.) Britton [Lamiaceae; Perillae Folium] |
| 97 | 13.92 | 314.079 | 314.0873 | 26.1 | 5,8-Dihydroxy-6,7-dimethoxyflavone | *Citrus aurantium* L. [Rutaceae; Aurantii Fructus] |
| 98 | 13.97 | 580.1792 | 580.1773 | -3.2 | Naringin | *Citrus aurantium* L. [Rutaceae; Aurantii Fructus] |
| 99 | 14.88 | 610.1897 | 610.1847 | -8.4 | Neohesperidin | *Citrus aurantium* L. [Rutaceae; Aurantii Fructus] |
| 100 | 15.07 | 396.1784 | 396.1874 | 20.3 | Cordifolioidyne B | *Codonopsis pilosula* (Franch.) Nannf. [Campanulaceae; Codonopsis Radix] |
| 101 | 15.18 | 610.1897 | 610.1844 | -8.9 | Hesperidin | *Citrus aurantium* L. [Rutaceae; Aurantii Fructus] |
| 102 | 15.47 | 356.1623 | 356.1698 | 20.8 | Gingerenone A | *Zingiber officinale* Roscoe [Zingiberaceae; Zingiberis Rhizoma] |
| 103 | 15.57 | 330.0739 | 330.082 | 24.3 | Viscidulin II | *Scutellaria baicalensis* Georgi [Lamiaceae; Scutellariae Radix] |
| 104 | 15.86 | 300.0633 | 300.0722 | 29.1 | 5,8,2'-Trihydroxy-7-methoxyflavone | *Scutellaria baicalensis* Georgi [Lamiaceae; Scutellariae Radix] |
| 105 | 15.98 | 400.2461 | 400.2567 | 23.7 | Atractyloside C | *Atractylodes lancea* (Thunb.) DC. [Asteraceae; Atractylodis Rhizoma] |
| 106 | 16 | 194.0579 | 194.0596 | 7.1 | Ferulic acid | *Codonopsis pilosula* (Franch.) Nannf. [Campanulaceae; Codonopsis Radix] |
| 107 | 16.49 | 460.1005 | 460.1046 | 8.1 | Baicalin | *Scutellaria baicalensis* Georgi [Lamiaceae; Scutellariae Radix] |
| 108 | 16.83 | 267.147 | 267.1443 | -9 | Codonopsine | *Codonopsis pilosula* (Franch.) Nannf. [Campanulaceae; Codonopsis Radix] |
| 109 | 16.9 | 314.1729 | 314.1679 | -13.9 | Perilloside A | *Perilla frutescens* (L.) Britton [Lamiaceae; Perillae Folium] |
| 110 | 16.93 | 448.1005 | 448.1094 | 19.8 | Luteolin-7-O-glucoside | *Perilla frutescens* (L.) Britton [Lamiaceae; Perillae Folium] |
| 111 | 17.78 | 460.1005 | 460.1088 | 18 | Oroxindin | *Scutellaria baicalensis* Georgi [Lamiaceae; Scutellariae Radix] |
| 112 | 17.97 | 611.1612 | 611.1751 | 21.2 | Cyanin | *Perilla frutescens* (L.) Britton [Lamiaceae; Perillae Folium] |
| 113 | 17.97 | 448.2308 | 448.2351 | 8.6 | Atractyloside A | *Atractylodes lancea* (Thunb.) DC. [Asteraceae; Atractylodis Rhizoma] |
| 114 | 18.03 | 260.1048 | 260.1143 | 36.2 | Meranzin | *Citrus aurantium* L. [Rutaceae; Aurantii Fructus] |
| 115 | 18.07 | 357.1787 | 357.1706 | -22.8 | 7-Acetylintermedine N-oxide | *Eupatorium fortunei* Turcz. [Asteraceae; Eupatorii Herba] |
| 116 | 18.3 | 274.1568 | 274.1517 | -16.6 | 3β-Acetoxyatractylone | *Atractylodes lancea* (Thunb.) DC. [Asteraceae; Atractylodis Rhizoma] |
| 117 | 18.69 | 367.1631 | 367.1582 | -13.4 | Jacobine N-oxide | *Eupatorium fortunei* Turcz. [Asteraceae; Eupatorii Herba] |
| 118 | 18.92 | 580.1792 | 580.1724 | -10.8 | Narirutin | *Citrus aurantium* L. [Rutaceae; Aurantii Fructus] |
| 119 | 19.49 | 284.0684 | 284.0775 | 31.5 | Wogonin | *Scutellaria baicalensis* Georgi [Lamiaceae; Scutellariae Radix] |
| 120 | 19.92 | 276.1725 | 276.1814 | 31.9 | 6-Shogaol | *Zingiber officinale* Roscoe [Zingiberaceae; Zingiberis Rhizoma] |
| 121 | 20.02 | 374.1001 | 374.1065 | 16.9 | Skullcapflavone II | *Scutellaria baicalensis* Georgi [Lamiaceae; Scutellariae Radix] |
| 122 | 20.05 | 278.1881 | 278.174 | -44 | Paradol | *Zingiber officinale* Roscoe [Zingiberaceae; Zingiberis Rhizoma] |
| 123 | 20.05 | 269.1263 | 269.1132 | -48.9 | Codonopsinol A | *Codonopsis pilosula* (Franch.) Nannf. [Campanulaceae; Codonopsis Radix] |
| 124 | 20.15 | 284.0684 | 284.0774 | 31.2 | Acacetin | *Coptis chinensis* Franch. [Ranunculaceae; Coptidis Rhizoma] |
| 125 | 20.17 | 341.1474 | 341.1563 | 25.8 | Monocrotaline N-oxide | *Atractylodes lancea* (Thunb.) DC. [Asteraceae; Atractylodis Rhizoma] |
| 126 | 20.21 | 357.0767 | 357.0773 | 1.5 | Berberrubine | *Coptis chinensis* Franch. [Ranunculaceae; Coptidis Rhizoma] |
| 127 | 20.29 | 413.2049 | 413.2022 | -6.8 | Echimidine N-oxide | *Eupatorium fortunei* Turcz. [Asteraceae; Eupatorii Herba] |
| 128 | 20.49 | 492.1267 | 492.1321 | 10.9 | Eupatolin | *Eupatorium fortunei* Turcz. [Asteraceae; Eupatorii Herba] |
| 129 | 20.95 | 344.0896 | 344.0969 | 21.1 | Tenaxin I | *Scutellaria baicalensis* Georgi [Lamiaceae; Scutellariae Radix] |
| 130 | 21.42 | 472.2097 | 472.2129 | 6.1 | Obacunoic acid | *Coptis chinensis* Franch. [Ranunculaceae; Coptidis Rhizoma] |
| 131 | 21.83 | 416.241 | 416.2473 | 13.9 | Atractyloside G | *Atractylodes lancea* (Thunb.) DC. [Asteraceae; Atractylodis Rhizoma] |
| 132 | 21.97 | 470.194 | 470.1973 | 6.3 | Limonin | *Citrus aurantium* L. [Rutaceae; Aurantii Fructus] |
| 133 | 21.98 | 514.2202 | 514.2211 | 1.5 | Nomilin | *Citrus aurantium* L. [Rutaceae; Aurantii Fructus] |
| 134 | 22.13 | 292.1674 | 292.1823 | 44.1 | Gingerdione | *Zingiber officinale* Roscoe [Zingiberaceae; Zingiberis Rhizoma] |
| 135 | 22.16 | 307.2147 | 307.2095 | -17 | Dihydrocapsaicin | *Zingiber officinale* Roscoe [Zingiberaceae; Zingiberis Rhizoma] |
| 136 | 22.17 | 1018.3529 | 1018.3946 | 39.2 | Tangshenoside IV | *Codonopsis pilosula* (Franch.) Nannf. [Campanulaceae; Codonopsis Radix] |
| 137 | 22.32 | 294.1831 | 294.192 | 30.2 | 6-Gingerol | *Zingiber officinale* Roscoe [Zingiberaceae; Zingiberis Rhizoma] |
| 138 | 22.34 | 594.2729 | 594.2802 | 11.3 | Obamegine | *Coptis chinensis* Franch. [Ranunculaceae; Coptidis Rhizoma] |
| 139 | 22.59 | 206.2034 | 206.2015 | -9.5 | (Z)-Caryophyllene | *Atractylodes lancea* (Thunb.) DC. [Asteraceae; Atractylodis Rhizoma] |
| 140 | 22.66 | 266.1518 | 266.163 | 38.7 | Magnograndiolide | *Scutellaria baicalensis* Georgi [Lamiaceae; Scutellariae Radix] |
| 141 | 22.97 | 676.367 | 676.3635 | -5 | Gingerglycolipid A | *Zingiber officinale* Roscoe [Zingiberaceae; Zingiberis Rhizoma] |
| 142 | 23.32 | 316.1885 | 316.18 | -23.8 | Perilloside C | *Perilla frutescens* (L.) Britton [Lamiaceae; Perillae Folium] |
| 143 | 23.65 | 304.2038 | 304.2121 | 27 | 8-Shogaol | *Zingiber officinale* Roscoe [Zingiberaceae; Zingiberis Rhizoma] |
| 144 | 23.69 | 232.1463 | 232.1566 | 43.9 | 3β-Hydroxyatractylone | *Atractylodes lancea* (Thunb.) DC. [Asteraceae; Atractylodis Rhizoma] |
| 145 | 23.94 | 232.1463 | 232.1567 | 44.3 | Atractylenolide II | *Atractylodes lancea* (Thunb.) DC. [Asteraceae; Atractylodis Rhizoma] |
| 146 | 24.38 | 264.2453 | 264.2308 | -46.8 | Linolenyl alcohol | *Perilla frutescens* (L.) Britton [Lamiaceae; Perillae Folium] |
| 147 | 24.98 | 220.219 | 220.2165 | -9.9 | Elemol | *Perilla frutescens* (L.) Britton [Lamiaceae; Perillae Folium] |
| 148 | 24.98 | 220.1827 | 220.171 | -44.3 | (-)-Epoxycaryophyllene | *Atractylodes lancea* (Thunb.) DC. [Asteraceae; Atractylodis Rhizoma] |
| 149 | 25.73 | 454.1991 | 454.2068 | 16.9 | Obacunone | *Coptis chinensis* Franch. [Ranunculaceae; Coptidis Rhizoma] |
| 150 | 26.24 | 248.1412 | 248.1512 | 39.8 | Atractylenolide III | *Atractylodes lancea* (Thunb.) DC. [Asteraceae; Atractylodis Rhizoma] |
| 151 | 26.38 | 380.1835 | 380.1771 | -16.9 | Cordifolioidyne C | *Codonopsis pilosula* (Franch.) Nannf. [Campanulaceae; Codonopsis Radix] |
| 152 | 26.52 | 349.1525 | 349.1661 | 34.4 | Erucifoline | *Eupatorium fortunei* Turcz. [Asteraceae; Eupatorii Herba] |
| 153 | 27.07 | 394.2355 | 394.2461 | 26.9 | 6-Methylgingediacetate | *Zingiber officinale* Roscoe [Zingiberaceae; Zingiberis Rhizoma] |
| 154 | 30.29 | 381.2303 | 381.2445 | 36.9 | Obscurine | *Coptis chinensis* Franch. [Ranunculaceae; Coptidis Rhizoma] |
| 155 | 30.66 | 624.3199 | 624.3509 | 47.9 | Neferine | *Coptis chinensis* Franch. [Ranunculaceae; Coptidis Rhizoma] |
| 156 | 32.14 | 608.2886 | 608.2623 | -43.2 | Demethyltetrandrine | *Coptis chinensis* Franch. [Ranunculaceae; Coptidis Rhizoma] |
| 157 | 32.58 | 456.3603 | 456.364 | 7.4 | Oleanolic acid | *Eupatorium fortunei* Turcz. [Asteraceae; Eupatorii Herba] |
| 158 | 34.24 | 622.3042 | 622.3106 | 10.1 | Tetrandrine | *Coptis chinensis* Franch. [Ranunculaceae; Coptidis Rhizoma] |
| 159 | 34.87 | 182.0731 | 182.0817 | 46.9 | Atractylodin | *Atractylodes lancea* (Thunb.) DC. [Asteraceae; Atractylodis Rhizoma] |
| 160 | 35.56 | 426.3861 | 426.3808 | -12.7 | Lupeol | *Codonopsis pilosula* (Franch.) Nannf. [Campanulaceae; Codonopsis Radix] |
| 161 | 36.23 | 194.0942 | 194.0952 | 4.2 | Zingerone | *Zingiber officinale* Roscoe [Zingiberaceae; Zingiberis Rhizoma] |
| 162 | 36.37 | 562.2989 | 562.3 | 1.9 | Atractyloside D | *Atractylodes lancea* (Thunb.) DC. [Asteraceae; Atractylodis Rhizoma] |
| 163 | 36.38 | 342.1705 | 342.1755 | 13.6 | Phellodendrine | *Coptis chinensis* Franch. [Ranunculaceae; Coptidis Rhizoma] |
| 164 | 36.44 | 600.4178 | 600.4231 | 8.8 | Neoxanthin | *Perilla frutescens* (L.) Britton [Lamiaceae; Perillae Folium] |

**Supplementary Table 3**

Atypical Symptoms Scoring Table.

| Atypical Symptoms Scoring | | |
| --- | --- | --- |
| Atypical Symptoms | Frequency Score | Severity Score |
| Coughing |  |  |
| Asthma |  |  |
| Total Score |  |  |
| Scoring is based on both the frequency and severity of symptoms:   - Frequency Score:   None: 0 points  Less than 1 day per week: 1 point  1 day per week: 2 points  2–3 days per week: 3 points  4–5 days per week: 4 points  6–7 days per week: 5 points   - Severity Score:   Symptoms are mild and unnoticeable without a doctor's reminder: 1 point  Symptoms are moderate, affecting daily life and requiring occasional medication: 3 points  Symptoms are severe, significantly affecting daily life and necessitating long-term medication: 5 points  Between mild and moderate: 2 points  Between moderate and severe: 4 points | | |

**Supplementary Table 4**

Spleen Deficiency and Damp-Heat Syndrome Scoring Table.

| Spleen Deficiency and Damp-Heat Syndrome Scoring | |
| --- | --- |
| Postprandial acid reflux | □ 0 None □ 2 Mild □ 4 Moderate □ 6 Severe |
| Bloating | □ 0 None □ 2 Mild □ 4 Moderate □ 6 Severe |
| Burning pain in the upper abdomen | □ 0 None □ 1 Mild □ 2 Moderate □ 3 Severe |
| Chest discomfort | □ 0 None □ 1 Mild □ 2 Moderate □ 3 Severe |
| Loss of appetite (anorexia) | □ 0 None □ 1 Mild □ 2 Moderate □ 3 Severe |
| Fatigue and weakness | □ 0 None □ 1 Mild □ 2 Moderate □ 3 Severe |
| Loose stools (diarrhea) | □ 0 None □ 1 Mild □ 2 Moderate □ 3 Severe |
| Constipation (difficult defecation) | □ 0 None □ 1 Mild □ 2 Moderate □ 3 Severe |

**Supplementary Table 5**

Demographic and Medication Adherence Profiles of FAS Participants.

|  | Placebo group | JQ granules group | P-value |
| --- | --- | --- | --- |
| **Age^a^** |  |  | 0.0973 |
| N (Nmiss) | 39 (0) | 39 (0) |  |
| Mean (SD) | 48.49 ± 16.22 | 42.64 ± 14.49 |  |
| **Height^a^** |  |  | 0.5703 |
| N (Nmiss) | 39 (0) | 39 (0) |  |
| Mean (SD) | 165.6 ± 7.56 | 166.58 ± 7.54 |  |
| **Weight^a^** |  |  | 0.2458 |
| N (Nmiss) | 39 (0) | 39 (0) |  |
| Mean (SD) | 60.73 ± 9.85 | 63.86 ± 13.49 |  |
| **Medication Compliance^a^** |  |  | 0.4527 |
| N (Nmiss) | 39 (0) | 39 (0) |  |
| Mean (SD) | 96.95 ± 11.35 | 94.25 ± 19.26 |  |

^a^ t-test

Abbreviations: JQ: JianpiQinghua; N: Number of participants; Nmiss: Number of missing values; SD: Standard Deviation.

**Supplementary Table 6**

Concomitant Medications and Medical History of Patients in the FAS.

|  | Placebo group (N=39) | JQ granules group (N=39) | P-value |
| --- | --- | --- | --- |
| **Prior Concomitant Medications^a^** |  |  | 0.7613 |
| Instances | 8 | 8 |  |
| Cases (Percentage) | 7 (17.95%) | 6 (15.38%) |  |
| **Concomitant Medications During Study^b^** |  |  | 1 |
| Instances | 7 | 7 |  |
| Cases (Percentage) | 2 (5.13%) | 2 (5.13%) |  |
| **Medical History^b^** |  |  |  |
| None | 36 (92.31%) | 37 (94.87%) | 1 |
| Yes | 3 (7.69%) | 2 (5.13%) |  |
| **Allergy History^b^** |  |  |  |
| None | 34 (87.18%) | 34 (87.18%) | 1 |
| Yes | 5 (12.82%) | 5 (12.82%) |  |

**^a^**Chi-square test

**^b^**Fisher's exact test

Abbreviations: JQ: JianpiQinghua; N: Number of participants.

**Supplementary Table 7**

Analysis of Cough Frequency and Severity Scores.

|  | Placebo group | JQ granules group | P-value |
| --- | --- | --- | --- |
| **Cough Frequency** |  |  |  |
| **Baseline^a^** | 1.13 ± 1.81 | 1.62 ± 1.98 | 0.20217 |
| N (Nmiss) | 39 (0) | 39 (0) |  |
| **Treatment Week 4^a^** | 0.84 ± 1.72 | 0.62 ± 1.34 | 0.93912 |
| N (Nmiss) | 38 (1) | 37 (2) |  |
| **Follow-up Week 4^a^** | 0.5 ± 1.35 | 0.62 ± 1.32 | 0.41251 |
| N (Nmiss) | 38 (1) | 37 (2) |  |
| **4 Weeks of Treatment vs Baseline^b^** | -0.32 ± 1.74 | -0.95 ± 2.08 | 0.1064 |
| N (Nmiss) | 38 (1) | 37 (2) |  |
| P-value^c^ | 0.27085 | 0.00892 |  |
| **4 Weeks of Follow-up vs Baseline^b^** | -0.66 ± 1.55 | -0.95 ± 2.15 | 0.4352 |
| N (Nmiss) | 38 (1) | 37 (2) |  |
| P-value^c^ | 0.01263 | 0.01102 |  |
| **Cough Severity** |  |  |  |
| **Baseline^a^** | 0.51 ± 0.76 | 0.79 ± 1.03 | 0.23802 |
| N (Nmiss) | 39 (0) | 39 (0) |  |
| **Treatment Week 4^a^** | 0.34 ± 0.78 | 0.3 ± 0.52 | 0.81206 |
| N (Nmiss) | 38 (1) | 37 (2) |  |
| **Follow-up Week 4^a^** | 0.21 ± 0.47 | 0.35 ± 0.63 | 0.34139 |
| N (Nmiss) | 38 (1) | 37 (2) |  |
| **4 Weeks of Treatment vs Baseline^b^** | -0.18 ± 0.95 | -0.46 ± 0.93 | 0.1578 |
| N (Nmiss) | 38 (1) | 37 (2) |  |
| P-value^c^ | 0.24176 | 0.00484 |  |
| **4 Weeks of Follow-up vs Baseline^b^** | -0.32 ± 0.74 | -0.41 ± 0.98 | 0.58 |
| N (Nmiss) | 38 (1) | 37 (2) |  |
| P-value^c^ | 0.01225 | 0.01696 |  |

**^a^** Rank-sum test

^b^ Analysis of Covariance

^c^ Paired rank-sum test within group

Abbreviations: JQ: JianpiQinghua; N: Number of participants; Nmiss: Number of missing values.

**Supplementary Table 8**

Analysis of Asthma Frequency and Severity Scores.

|  | Placebo group | JQ granules group | P-value |
| --- | --- | --- | --- |
| **Asthma Frequency** |  |  |  |
| **Baseline^a^** | 0.23 ± 0.71 | 0.33 ± 0.93 | 0.70502 |
| N (Nmiss) | 39 (0) | 39 (0) |  |
| **Treatment Week 4^a^** | 0.29 ± 1.14 | 0.05 ± 0.23 | 0.64142 |
| N (Nmiss) | 38 (1) | 37 (2) |  |
| **Follow-up Week 4^a^** | 0.26 ± 1.13 | 0 ± 0 | 0.16574 |
| N (Nmiss) | 38 (1) | 37 (2) |  |
| **4 Weeks of Treatment vs Baseline^b^** | 0.05±1.06 | -0.22±0.89 | 0.1827 |
| N (Nmiss) | 38 (1) | 37 (2) |  |
| P-value^c^ | 0.76215 | 0.14647 |  |
| **4 Weeks of Follow-up vs Baseline^b^** | 0.03 ± 1.05 | -0.27 ± 0.84 | 0.1399 |
| N (Nmiss) | 38 (1) | 37 (2) |  |
| P-value^c^ | 0.87833 | 0.05763 |  |
| **Asthma Severity** |  |  |  |
| **Baseline^a^** | 0.23 ± 0.63 | 0.23 ± 0.67 | 1 |
| N (Nmiss) | 39 (0) | 39 (0) |  |
| **Treatment Week 4^a^** | 0.08 ± 0.27 | 0.08 ± 0.28 | 0.98201 |
| N (Nmiss) | 38 (1) | 37 (2) |  |
| **Follow-up Week 4^a^** | 0.11 ± 0.39 | 0.03 ± 0.16 | 0.32047 |
| N (Nmiss) | 38 (1) | 37 (2) |  |
| **4 Weeks of Treatment vs Baseline^b^** | -0.16 ± 0.59 | -0.14 ± 0.71 | 1 |
| N (Nmiss) | 38 (1) | 37 (2) |  |
| P-value^c^ | 0.10974 | 0.25686 |  |
| **4 Weeks of Follow-up vs Baseline^b^** | -0.13 ± 0.58 | -0.19 ± 0.66 | 0.5471 |
| N (Nmiss) | 38 (1) | 37 (2) |  |
| P-value^c^ | 0.16855 | 0.0897 |  |

**^a^** Rank-sum test

^b^ Analysis of Covariance

^c^ Paired rank-sum test within group

Abbreviations: JQ: JianpiQinghua; N: Number of participants; Nmiss: Number of missing values.

**Supplementary Table 9**

Analysis of GERD-HRQL Scale Scores.

|  | Placebo group | JQ granules group | P-value |
| --- | --- | --- | --- |
| **GERD-HRQL Scale Scores** |  |  |  |
| **Baseline^a^** | 13.59 ± 8.04 | 14.85 ± 8.54 | 0.5054 |
| N (Nmiss) | 39 (0) | 39 (0) |  |
| **Treatment Week 4^a^** | 10.79 ± 8.01 | 10.32 ± 9.09 | 0.8147 |
| N (Nmiss) | 38 (1) | 37 (2) |  |
| **Follow-up Week 4^a^** | 9.84 ± 7.7 | 8.81 ± 8.74 | 0.5892 |
| N (Nmiss) | 38 (1) | 37 (2) |  |
| **4 Weeks of Treatment vs Baseline^b^** | -2.63 ± 4.94 | -4.41 ± 6.74 | 0.2078 |
| N (Nmiss) | 38 (1) | 37 (2) |  |
| P-value^c^ | 0.0022423 | 0.0003253 |  |
| **4 Weeks of Follow-up vs Baseline^b^** | -3.58 ± 5.73 | -5.92±7.35 | 0.1376 |
| N (Nmiss) | 38 (1) | 37 (2) |  |
| P-value^c^ | 0.0004486 | < 0.0001 |  |

**^a^** t-test

^b^ Analysis of Covariance

^c^ Paired t-test within group

Abbreviations: JQ: JianpiQinghua; N: Number of participants; Nmiss: Number of missing values.

**Supplementary Table 10**

Analysis of Dyspepsia, Bowel Irregularity, Psychological Mood, Reflux, Systemic Symptom, Social Functioning, Total Scores on the Chronic Gastrointestinal Disease PRO Scale.

|  | Placebo group | JQ granules group | P-value |
| --- | --- | --- | --- |
| Dyspepsia |  |  |  |
| **Baseline^a^** | 15.08 ± 8.21 | 14.49 ± 7.44 | 0.50531 |
| N (Nmiss) | 39 (0) | 39 (0) |  |
| **Treatment Week 4^a^** | 10.87 ± 8.61 | 10.35 ± 8.38 | 0.7662 |
| N (Nmiss) | 38 (1) | 37 (2) |  |
| **Follow-up Week 4^a^** | 10.34 ± 8.72 | 9.68 ± 7.8 | 0.79865 |
| N (Nmiss) | 38 (1) | 37 (2) |  |
| **4 Weeks of Treatment vs Baseline^a^** | -4.18 ± 5.23 | -4.16 ± 4.64 | 0.49269 |
| N (Nmiss) | 38 (1) | 37 (2) |  |
| P-value^b^ | < 0.0001 | < 0.0001 |  |
| **4 Weeks of Follow-up vs Baseline^a^** | -4.71 ± 6.11 | -4.84 ± 5.41 | 0.52384 |
| N (Nmiss) | 38 (1) | 37 (2) |  |
| P-value^b^ | < 0.0001 | < 0.0001 |  |
| Bowel Irregularity |  |  |  |
| **Baseline^a^** | 6.49 ± 5.08 | 6.08 ± 3.9 | 0.8804 |
| N (Nmiss) | 39 (0) | 39 (0) |  |
| **Treatment Week 4^a^** | 4.37 ± 4.52 | 4.16 ± 3.44 | 0.74453 |
| N (Nmiss) | 38 (1) | 37 (2) |  |
| **Follow-up Week 4^a^** | 4.03 ± 5.18 | 3.08 ± 3.45 | 0.91453 |
| N (Nmiss) | 38 (1) | 37 (2) |  |
| **4 Weeks of Treatment vs Baseline^a^** | -2.16 ± 3.33 | -1.86 ± 3.3 | 0.93565 |
| N (Nmiss) | 38 (1) | 37 (2) |  |
| P-value^b^ | < 0.0001 | < 0.0001 |  |
| **4 Weeks of Follow-up vs Baseline^a^** | -2.5 ± 4.16 | -2.95 ± 4.36 | 0.53576 |
| N (Nmiss) | 38 (1) | 37 (2) |  |
| P-value^b^ | 0.00019 | < 0.0001 |  |
| Psychological Mood |  |  |  |
| **Baseline^a^** | 3.87 ± 3.54 | 3.92 ± 3.37 | 0.82442 |
| N (Nmiss) | 39 (0) | 39 (0) |  |
| **Treatment Week 4^a^** | 2.95 ± 2.89 | 2.68 ± 2.93 | 0.59515 |
| N (Nmiss) | 38 (1) | 37 (2) |  |
| **Follow-up Week 4^a^** | 2.47 ± 2.46 | 2.32 ± 2.94 | 0.54918 |
| N (Nmiss) | 38 (1) | 37 (2) |  |
| **4 Weeks of Treatment vs Baseline^a^** | -1 ± 1.66 | -1.16 ± 1.72 | 0.62066 |
| N (Nmiss) | 38 (1) | 37 (2) |  |
| P-value^b^ | 0.00072 | < 0.0001 |  |
| **4 Weeks of Follow-up vs Baseline^a^** | -1.47 ± 2.54 | -1.51 ± 2.13 | 0.59162 |
| N (Nmiss) | 38 (1) | 37 (2) |  |
| P-value^b^ | 0.00067 | < 0.0001 |  |
| Reflux |  |  |  |
| **Baseline^a^** | 7.44 ± 2.58 | 7.85 ± 3.39 | 0.82886 |
| N (Nmiss) | 39 (0) | 39 (0) |  |
| **Treatment Week 4^a^** | 6.61 ± 3.43 | 5.92 ± 4.09 | 0.26152 |
| N (Nmiss) | 38 (1) | 37 (2) |  |
| **Follow-up Week 4^a^** | 6.5 ± 3.84 | 5.54 ± 4.53 | 0.20518 |
| N (Nmiss) | 38 (1) | 37 (2) |  |
| **4 Weeks of Treatment vs Baseline^a^** | -0.84 ± 3.01 | -1.97 ± 3.53 | 0.06735 |
| N (Nmiss) | 38 (1) | 37 (2) |  |
| P-value^b^ | 0.0486 | 0.00046 |  |
| **4 Weeks of Follow-up vs Baseline^a^** | -0.95 ± 3.62 | -2.35 ± 4.2 | 0.12028 |
| N (Nmiss) | 38 (1) | 37 (2) |  |
| P-value^b^ | 0.08239 | 0.00108 |  |
| Systemic Symptom |  |  |  |
| **Baseline^a^** | 3.67 ± 2.77 | 3.13 ± 2.6 | 0.42812 |
| N (Nmiss) | 39 (0) | 39 (0) |  |
| **Treatment Week 4^a^** | 3.18±2.58 | 2.41 ± 2.44 | 0.19228 |
| N (Nmiss) | 38 (1) | 37 (2) |  |
| **Follow-up Week 4^a^** | 2.71 ± 2.94 | 2.05 ± 2.34 | 0.76586 |
| N (Nmiss) | 38 (1) | 37 (2) |  |
| **4 Weeks of Treatment vs Baseline^a^** | -0.55 ± 1.41 | -0.54 ± 2.16 | 0.74495 |
| N (Nmiss) | 38 (1) | 37 (2) |  |
| P-value^b^ | 0.02233 | 0.19602 |  |
| **4 Weeks of Follow-up vs Baseline^a^** | -1.03 ± 1.81 | -0.89 ± 2.25 | 0.88326 |
| N (Nmiss) | 38 (1) | 37 (2) |  |
| P-value^b^ | 0.00028 | 0.01548 |  |
| Social Functioning |  |  |  |
| **Baseline^a^** | 1.31±1.28 | 1.13±1.94 | 0.10898 |
| N (Nmiss) | 39 (0) | 39 (0) |  |
| **Treatment Week 4^a^** | 1.08±1.42 | 0.62±1.38 | 0.03742 |
| N (Nmiss) | 38 (1) | 37 (2) |  |
| **Follow-up Week 4^a^** | 0.79±1.17 | 0.54±1.52 | 0.03399 |
| N (Nmiss) | 38 (1) | 37 (2) |  |
| **4 Weeks of Treatment vs Baseline^a^** | -0.21±1.14 | -0.43±0.87 | 0.36987 |
| N (Nmiss) | 38 (1) | 37 (2) |  |
| P-value^b^ | 0.23773 | 0.00586 |  |
| **4 Weeks of Follow-up vs Baseline^a^** | -0.5±1.06 | -0.51±1.04 | 0.93214 |
| N (Nmiss) | 38 (1) | 37 (2) |  |
| P-value^b^ | 0.00201 | 0.00391 |  |
| Total Scores |  |  |  |
| **Baseline^c^** | 38.56 ± 18.18 | 37.49 ± 18.72 | 0.7973 |
| N (Nmiss) | 39 (0) | 39 (0) |  |
| **Treatment Week 4^c^** | 29.61 ± 19.3 | 26.81 ± 20.12 | 0.5412 |
| N (Nmiss) | 38 (1) | 37 (2) |  |
| **Follow-up Week 4^c^** | 27.39 ± 20.17 | 23.76 ± 19.12 | 0.4256 |
| N (Nmiss) | 38 (1) | 37 (2) |  |
| **4 Weeks of Treatment vs Baseline^d^** | -9.11 ± 10.47 | -10.38 ± 11.72 | 0.5815 |
| N (Nmiss) | 38 (1) | 37 (2) |  |
| P-value^e^ | < 0.0001 | < 0.0001 |  |
| **4 Weeks of Follow-up vs Baseline^d^** | -11.32 ± 13.4 | -13.43 ± 14.38 | 0.4862 |
| N (Nmiss) | 38 (1) | 37 (2) |  |
| P-value^e^ | < 0.0001 | < 0.0001 |  |

**^a^** Rank-sum test

^b^ Paired rank-sum test within group

**^c^** t-test

^d^ Analysis of Covariance

^e^ Paired t-test within group

Abbreviations: JQ: JianpiQinghua; N: Number of participants; Nmiss: Number of missing values.

**Supplementary Table 11**

List of Adverse Events.

| Center Number | Subject Number | Group | Adverse Event Name | Start Date | End Date | Adverse Event Severity | Serious Adverse Event | Action Taken for Study Drug | Outcome of Adverse Event | Symptomatic Treatment for Adverse Event | Relation to Study Drug | Patient Withdrawal Due to Event | Description and Management of Adverse Reaction/Event (including symptoms, signs, clinical tests, etc.) |
| --- | --- | --- | --- | --- | --- | --- | --- | --- | --- | --- | --- | --- | --- |
| 1 | 22 | Experimental | Lip Blisters | 2021-12-10 | 2021-12-17 | Severe | No | Dose Reduction | Relieved | No | Suspicious | No | Lip blisters; reduced study drug |
| 1 | 22 | Experimental | Lip Blisters with Excessive Phlegm | 2021-12-20 | 2021-12-30 | Mild | No | Discontinuation | Relieved | Yes | Suspicious | Yes | Excessive phlegm, white in color, thick, difficult to expectorate, accompanied by cough, lip blisters |
